# Calcium-dependent redox signaling connects mitochondrial remodeling with PAD-associated NETosis during respiratory mycoplasma infection

**DOI:** 10.1016/j.redox.2026.104316

**Published:** 2026-07-18

**Authors:** Shun Wang, Weiqi Liu, Fuhua Gu, Jian Wang, Yuquan Guo, Liyang Guo, Yifan Li, Kexin Wang, Jie Zhang, Yecheng Yao, Zhiyong Wu, Jichang Li

**Affiliations:** a College of Veterinary Medicine, Northeast Agricultural University, 600 Changjiang Road, Xiangfang District, Harbin, 150030, PR China; b Heilongjiang Key Laboratory for Animal Disease Control and Pharmaceutical Development, 600 Changjiang Road, Xiangfang District, Harbin, 150030, PR China

**Keywords:** Redox signaling, Calcium signaling, Mitophagy, Neutrophil extracellular traps, *Mycoplasma gallisepticum*

## Abstract

Neutrophil extracellular trap (NET) formation is controlled by redox signaling and mitochondrial stress, but the connection between pathogen-induced Ca^2+^ influx, mitochondrial remodeling, and PAD-associated chromatin execution remains insufficiently defined. Using *Mycoplasma gallisepticum* (MG) as a model of respiratory mycoplasma infection, we examined how pathogen-activated redox signaling modulates NET formation and the subsequent fate of extracellular DNA. In neutrophils, MG promoted NET formation, although visible trap deposition was partly obscured by MG-associated nuclease activity. Early proteomic analysis indicated enrichment of calcium signaling, ROS-related pathways, autophagy/mitophagy, lysosome/phagosome programs, and MAPK-linked responses. Mechanistically, MG-induced NETosis was mediated through involved a Ca^2+^/ROS-associated program, in which mitochondrial dysfunction and mitophagy-related remodeling facilitated PAD3 nuclear redistribution, histone citrullination, and extracellular DNA release. In agreement with a supportive rather than exclusive function, mitophagy activation enhanced NET-associated responses, whereas mitophagy inhibition weakened but did not completely prevent MG-induced NET release. MG-associated nuclease activity digested extracellular traps, enabled MG to acquire DNA signals derived from digested trap structures, and lowered NET-dependent inflammatory activation in recipient macrophages and epithelial cells. *In vivo*, MG infection caused local NET-related responses and systemic neutrophil priming, linked to mainly associated with ROS/MAPK activation rather than prolonged mitophagy-related alterations. MG–*Escherichia coli* co-infection intensified inflammatory pathology, whereas DNase I produced partial protection. These results support a context-dependent model in which MG stimulates Ca^2+^-dependent redox signaling and mitochondrial remodeling, thereby contributing to PAD-associated NETosis, while pathogen nuclease activity modifies extracellular NET DNA fate and downstream inflammatory pathology.

## Highlights

• Mycoplasma nuclease degrades NETs and facilitates access to DNA-derived products.
• Ca^2+^/ROS–mitophagy–PAD signaling licenses pathogen-induced NET release.
• ROS/MAPK-associated priming contributes to enhanced NET release in *Mycoplasma gallisepticum*-exposed neutrophil.

## Graphical abstract

Flow chart and proposed model of MG-induced NETosis and extracellular NET DNA remodeling. Image created by Figdraw (ID: IWOIA088c4 and YSYOT88c8c).

**Figure:**
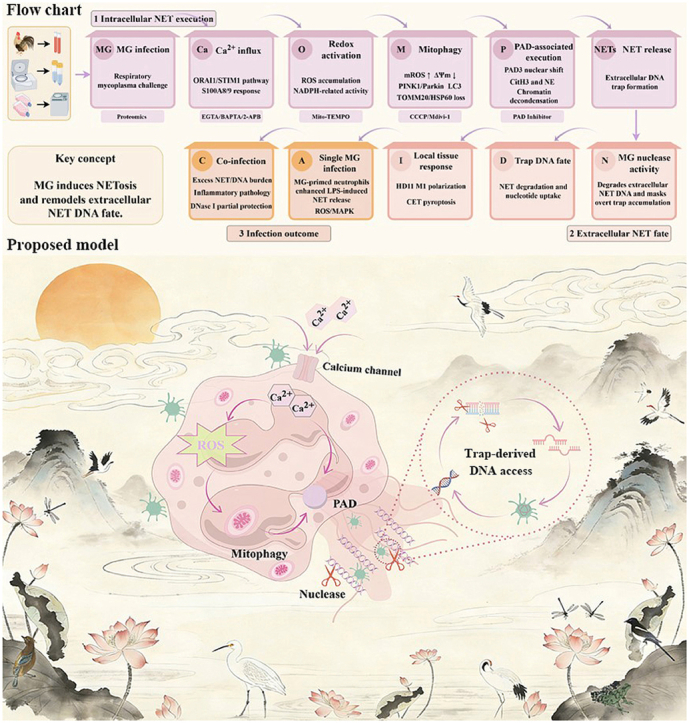

## 1 Introduction

Neutrophils are among the earliest immune cells recruited during infection and form a frontline element of host defense [1,2]. At infection sites, neutrophils limit invading pathogens through oxidative burst, degranulation, phagocytosis, and the generation of neutrophil extracellular traps (NETs) [1]. NETs are extracellular chromatin-based networks decorated with histones, granular enzymes, antimicrobial proteins, and inflammatory mediators, allowing them to immobilize pathogens and strengthen local host defense [3]. However, NETs are not solely protective. Excessive or prolonged NET accumulation can expose extracellular DNA, histones, proteases, and oxidant-associated mediators that intensify inflammation and contribute to tissue pathology [4]. Therefore, clarifying how pathogen-induced NET formation is initiated, executed, and remodeled after release is important for understanding both antimicrobial defense and infection-associated inflammatory injury.

NET formation is a controlled chromatin-externalization process that depends on the coordinated activation of ion signaling, oxidative responses, granule-associated proteins, and chromatin-modifying enzymes [1,5]. During neutrophil activation, cytosolic Ca^2+^ mobilization can support peptidylarginine deiminase (PAD)-family enzyme activity and histone citrullination, thereby weakening histone–DNA electrostatic interactions and facilitating chromatin decondensation [5,6]. In parallel, reactive oxygen species (ROS) produced through NADPH oxidase-dependent and mitochondrial pathways contribute to neutrophil activation and may facilitate granule protein mobilization, neutrophil elastase-associated histone processing, PAD-associated chromatin modification, and extracellular DNA release [7,8]. Autophagy-related remodeling has also been associated with NET formation, because classical PMA-induced NETosis requires both superoxide production and autophagy for intracellular chromatin decondensation and NET release [9,10]. However, the requirements for NADPH oxidase, myeloperoxidase, Ca^2+^ signaling, ROS production, and histone citrullination differ considerably according to the stimulus, indicating that NET execution is strongly context dependent [1,11]. Thus, although Ca^2+^, ROS, autophagy-related remodeling, granule proteins, and PAD-mediated chromatin modification are widely accepted components of NET formation, how pathogens integrate these modules to initiate and execute NET release remains not fully understood.

Ca^2+^ signaling represents a possible entry point for this pathogen-specific regulation. At the stage of NET release, Ca^2+^ has been identified as a key ionic signal, because chelation of either extracellular or intracellular Ca^2+^ can greatly affect the extent of NET formation [12]. Mechanistically, Ca^2+^ entry through store-operated Ca^2+^ entry pathways involving ORAI/STIM-associated mechanisms can maintain intracellular Ca^2+^ signals and support NADPH oxidase-associated ROS production [13,14]. In addition, Ca^2+^-dependent pathways, including calcium-activated K^+^ channels and Akt-associated signaling, have been connected with mitochondrial ROS generation in specific NET-inducing contexts [12,15]. Therefore, Ca^2+^ signaling may function as an upstream hub that connects pathogen sensing with ROS-associated oxidative activation, PAD-associated chromatin remodeling, and NET release.

Mitochondria are redox-active organelles that coordinate metabolic stress, ROS production, Ca^2+^ handling, and innate immune signaling [16]. Mitophagy serves as a selective mitochondrial quality-control process that eliminates damaged mitochondria and is often regulated by pathways such as the PINK1/Parkin axis [17]. In neutrophils, mitochondrial status is closely associated with effector functions, including oxidative burst, phagocytosis, and NET formation [18]. Although mitophagy has been proposed as a possible contributor to NET release [19,20], direct evidence defining its function during pathogen-induced NET formation remains limited. In particular, it remains unclear whether mitophagy-associated mitochondrial remodeling merely accompanies pathogen-induced ROS accumulation and mitochondrial stress, or actively connects ROS-associated oxidative activation with PAD-associated chromatin execution and NET release.

Mycoplasmas provide a useful pathogen model for investigating this issue. These cell-wall-deficient bacteria have undergone reductive evolution and strongly rely on host-derived metabolites, including nucleotide precursors [21]. They are commonly linked to chronic or persistent infection and can withstand neutrophil-mediated antimicrobial pressure [22,23]. In the context of NET biology, this resistance is especially notable. Previous studies have demonstrated that several mycoplasmas, including *Mycoplasma pneumoniae* [24], *Mycoplasma hyopneumoniae* [25], and *Mycoplasma bovis* [26], can stimulate NET release. For example, the MHO_0730 protein of *M. pneumoniae* can induce NET formation [27], although the exact mechanism remains unclear. Importantly, mycoplasmas can evade NET-mediated trapping and killing through nucleases such as MnuA [28], TatD [29], and Mhp597 [30], a process that has been widely examined. Although these studies emphasize the resistance of mycoplasmas to NETs, the molecular pathways through which mycoplasmas initiate NET formation remain poorly understood.

Among mycoplasmas, *Mycoplasma gallisepticum* (MG) is a representative respiratory pathogen that causes chronic airway infection, mucosal immune imbalance, persistent colonization, and increased susceptibility to mixed infection [31,32]. Previous studies have shown that chicken neutrophils can release NETs in response to different stimuli, including parasite-associated stimulation [33], fungal toxins [34], and environmental toxins [35], thereby contributing to host immune regulation. For example, fumonisin B1 suppresses zymosan-induced NET formation by lowering ROS levels in chicken neutrophils [36]. In addition, selenoprotein S deficiency enhances NET-associated neutrophil recruitment in chicken aortic tissue and contributes to arteritis progression [37]. Our previous work further demonstrated that MG contains nuclease-associated virulence traits and can degrade NETs, thereby decreasing local antimicrobial pressure [29]. However, the manner in which mycoplasma infection, particularly MG infection, triggers NET release and remodels extracellular trap fate remains poorly defined.

Here, MG infection was used as a pathogen model to investigate how MG-induced Ca^2+^/ROS signaling is connected with mitochondrial remodeling, PAD-related NET execution, and the fate of extracellular traps. MG was found to induce extracellular Ca^2+^ influx and ROS accumulation, followed by mitochondrial dysfunction, mitochondrial ROS buildup, and mitophagy-related remodeling. This mitochondrial remodeling contributed to nuclear redistribution of PAD3, histone citrullination, and NET release, whereas nuclease activity associated with MG degraded extracellular traps and changed the inflammatory effects of trap-derived DNA. Overall, these findings indicate a two-level mechanism of MG–NET interaction: an intracellular Ca^2+^/ROS–mitochondrial remodeling pathway that supports NET execution and an extracellular nuclease-dependent process that modifies trap fate after release.

## 2 Materials and methods

Additional information regarding experimental grouping strategies, treatment protocols, antibody sources and working concentrations, reagent details, and expanded descriptions is presented in the Supplementary Materials.

### 2.1 Culture and detection of MG and *Escherichia coli*

The MG-R_low_ strain was generously provided by the Harbin Veterinary Research Institute, Chinese Academy of Agricultural Sciences, and was maintained in our laboratory. MG was cultured at 37 °C in mycoplasma medium (H910KJ; Yuanpei Biotechnology Co., Ltd., Shanghai, China) following previously reported procedures [29], and bacterial titers were estimated as color-changing units (CCU/mL). To detect MG in tissues, DNA extracted (AG21009, ACCURATE BIOTECHNOLOGY (HUNAN) CO.,LTD, ChangSha, China) from infected samples was used as the template for PCR amplification of the *mgc2* gene. The primer sequences were as follows: MG-mgc2-F, 5′-GGTCCTAATCCCCAACAAAGAAT-3′; and MG-mgc2-R, 5′-CTTGGTTGGTTCATATTAGGCATTT-3′. PCR amplicons were resolved on 1% agarose gels and visualized using standard procedures. To further determine tissue MG burden, absolute quantification was carried out using a standard curve generated from a recombinant plasmid harboring the *mgc2* gene, as described previously [38].

*Escherichia coli* JD37 (*E. coli* JD37) was a polymyxin E resistant strain that had been previously isolated in our laboratory from an MG-infected farm in Harbin, China (S-Fig. 13A). The presence of *E. coli* JD37 in tissues was assessed by PCR amplification of the *mcr-1* gene using DNA extracted from tissue samples. The primer sequences were as follows: *E. coli* JD37-MCR1-F, 5′-ATGATGCAGCATACTTCTGTGTG-3′; and *E. coli* JD37-MCR1-R, 5′-TCAGCGGATGAATGCGGTGC-3′. As previously reported [39], *E. coli* JD37 was cultured in LB medium, and tissue colonization was evaluated by plating tissue homogenates onto LB agar supplemented with polymyxin E (2 μg/mL), followed by enumeration of bacterial colonies.

### 2.2 Cell culture and treatments

#### 2.2.1 HD11 cells culture and treatments

HD11 cells (chicken macrophage cell line; HTX2259) were obtained from OTWO Biotech Co., Ltd. Cells were cultured in RPMI 1640 medium containing 10% fetal bovine serum and 1% penicillin streptomycin at 37 °C in a humidified atmosphere with 5% CO_2_.

To investigate the effects of NETs on HD11 cells, five experimental groups were established: control group (CG), MG group (400 MOI), MG (400 MOI) + NETs (50 ng/mL) group, MG (400 MOI) + Aurintricarboxylic acid (ATA, 25 μM) + NETs (50 ng/mL) group, and NETs (50 ng/mL) group. Once cell confluence about 70%–80%, cells were exposed to PBS, MG, and/or NETs for 4 h or 10 h according to the requirements of the downstream assays, after which they were harvested for further analyses.

#### 2.2.2 CET cell culture and treatments

Immortalized chicken embryo tracheal epithelial cells (CETs) and their corresponding specialized culture medium were purchased from Shanghai Jinyuan Biotechnology Co., Ltd. (cell line: JY-J1434; medium: JY-Y13809). CETs were maintained at 37 °C in a humidified incubator containing 5% CO_2_ according to the manufacturer's recommendations.

To evaluate the effects of NETs on CETs, cells were divided into five groups: control group (CG), MG group (300 MOI), MG (300 MOI) + NETs (400 ng/mL) group, MG (300 MOI) + ATA (25 μM) + NETs (400 ng/mL) group, and NETs (400 ng/mL) group. Depending on the downstream analyses, cells were treated for either 12 h or 24 h, before being collected for subsequent experiments.

#### 2.2.3 Chicken neutrophil culture and treatments

Chicken neutrophils were isolated using a commercial kit (LZS1098C, Tianjin Haoyang Biological Manufacture Co., Ltd., China) according to the manufacturer's instructions and were further purified using a previously described method to enhance population purity [29,40]. Purified neutrophils were seeded at densities of 2 × 10^6^, 5 × 10^5^, or 2 × 10^4^ cells per well in 6-well, 12-well, or 24-well plates, respectively, each fitted with poly-l-lysine coated coverslips of the corresponding size (J06002, J12002, and J24002; Shanghai Jing'an Biotechnology Co., Ltd.). The neutrophils were cultured in RPMI 1640 medium supplemented with 10% fetal bovine serum and allowed to adhere for 1 h at 37 °C under humidified conditions with 5% CO_2_. Subsequently, non-adherent cells were removed by gently washing the wells three times with prewarmed PBS. Fresh complete RPMI 1640 medium was then added before the application of subsequent treatments.

Because the conventional mammalian NET marker framework cannot be directly applied to the avian system where MPO and PAD4 proteins have not been identified in chicken neutrophils [41,42], NET formation was primarily evaluated by extracellular DNA release together with CitH3 and NE detection, whereas PAD3 was included as an additional PAD-family indicator associated with histone citrullination [43]. Accordingly, PAD3 should be regarded as a marker linked to PAD-dependent chromatin execution in this model, rather than the sole PAD enzyme responsible for MG-induced NETosis.

### 2.3 Animal experiments and treatments

Specific pathogen-free chicks were obtained at 1 day of age from the Animal Experimental Center of the Harbin Veterinary Research Institute, Chinese Academy of Agricultural Sciences. Following a 7-day acclimation period, the chicks were used in the subsequent animal studies. All animal procedures were conducted in accordance with the guidelines of the Laboratory Animal Ethics Committee of Northeastern Agricultural University (approved No.: NEAUEC20240313).

To investigate the effect of MG infection on neutrophil responsiveness *in vivo*, chicks were randomly allocated into two groups: control group (CG) and MG group (n = 15). In the MG group, infection was established by bilateral air sac injection of 0.2 mL MG suspension (1 × 10^9^ CCU/mL), combined with auxiliary intranasal inoculation for 3 consecutive days [31,44]. Chicks in the CG group received an equal volume of sterile saline using the same administration procedure. After treatment, chicks were anesthetized, blood samples were collected, and neutrophils were isolated. A portion of the isolated neutrophils was subsequently stimulated with LPS (5 μg/mL; L12280, Sigma-Aldrich) for 3 h to induce NET formation. The detailed experimental workflow is presented in Fig. 10A.

To assess the contribution of MG infection to neutrophil-associated pathology during mixed infection, chicks were randomly assigned to five groups (n = 15): CG, MG, *E. coli* JD37, MG + *E. coli* JD37, and MG + *E. coli* JD37 + DNase I. Chicks in the designated groups were challenged intraperitoneally with 0.2 mL E*. coli* JD37 suspension (2 × 10^9^ CFU/mL) [39]. DNase I (11284932001, Roche) was administered at a dose of 50 U per chick. Following anesthesia, blood samples were collected and submitted to the Clinical Laboratory of the Affiliated Animal Hospital of Northeast Agricultural University for analysis. Routine hematological indices were subsequently determined using a hematology analyzer. The detailed experimental scheme is illustrated in Fig. 11A.

### 2.4 NET isolation

NETs were isolated with light modifications from a previously reported method [45]. Briefly, after neutrophil stimulation with PMA or MG, culture supernatants were carefully removed, and NET-containing material attached to the coverslips was collected by gentle pipetting with ice-cold PBS. The recovered suspensions were centrifuged twice at 4 °C and 500 ×g for 10 min to eliminate intact cells and large cellular debris. The obtained supernatants were then centrifuged at 4 °C and 2000 ×g to further decrease possible contamination by residual MG organisms. After this preclearing step, the supernatants were centrifuged at 4 °C and 18000 × g for 15 min to obtain a NET-enriched pellet, which was resuspended in ice-cold PBS.

### 2.5 Quantification of NET-associated dsDNA

NET-associated dsDNA was measured using a dsDNA detection kit (12643ES60, Yeasen Biotechnology Co., Ltd.). Briefly, dsDNA standards were serially diluted in the working solution supplied with the kit, and fluorescence was recorded using a microplate reader (Ex/Em = 480/520 nm) to prepare a standard curve. For samples obtained from adherent culture systems, micrococcal nuclease (2 gel units; D7201S, Beyotime) was added where indicated and incubated at 37 °C for 30 min to release NET-associated DNA attached to the coverslips into the supernatant. The reaction was subsequently stopped by adding EDTA to a final concentration of 5 mM. Samples were centrifuged at 4 °C and 1000 ×g for 10 min, and the supernatants were collected. The recovered samples were mixed with the working solution, incubated for 2 min in darkness, and then assessed by fluorescence measurement to determine dsDNA content.

### 2.6 NET visualization

To support structural visualization of NETs, the nuclease inhibitor aurintricarboxylic acid (ATA; 25 μM; HY-124493, MCE) (S-Fig. 3) was applied to inhibit MG-associated nuclease activity in all assays involving NET visualization. ATA was used as a structure-preserving tool for NET visualization rather than as evidence that nuclease inhibition completely restores physiological NET accumulation [46,47]. This treatment was used in Sytox Green staining, scanning electron microscopy (SEM), confocal microscopy, and immunofluorescence assays. Briefly, ATA was added 5 min before MG stimulation to reduce extracellular DNA degradation by MG-associated nuclease activity and thereby maintain NET structures for visualization. For Sytox Green staining, after the indicated treatments, cells were carefully washed three times with prewarmed PBS and fixed with 4% paraformaldehyde for 15 min. Samples were then incubated with Sytox Green working solution (1 μM; C1181S, Beyotime) for 15 min in the dark, and NET-associated DNA signals were observed using an inverted fluorescence microscope.

### 2.7 Early proteomic profiling of MG-stimulated neutrophils

To define early proteomic alterations in chicken neutrophils after MG stimulation, neutrophils were divided into two groups: CG and MG group. Cells in the CG group were treated with an equal volume of PBS, whereas cells in the MG group were stimulated with MG at an MOI of 80 for 1 h. After treatment, culture media were immediately discarded, and cells were gently washed with ice-cold PBS to remove residual medium and unbound stimuli. Cell pellets were subsequently collected for proteomic analysis. Samples were sent to Sangon Biotech (Shanghai, China) for proteomic profiling. In brief, proteins were extracted, and analyzed by LC-MS/MS. MS/MS data were searched against the corresponding *Gallus gallus* protein database. Protein identification was controlled at a false discovery rate of <1%. Differentially regulated proteins were defined according to fold-change and statistical thresholds as indicated. GO and KEGG enrichment analyses were conducted using the annotated protein list.

### 2.8 Statistical analysis

Experimental data were presented as the mean ± standard deviation and were evaluated using normality (Kolmogorov-Smirnov) and homogeneity (Homogeneity of variance) tests. Intergroup differences were analyzed by one-way ANOVA (Duncan's test) using SPSS (version 24.0) software. Different superscript letters among groups indicate statistically significant differences (*p* < 0.05).

## 3 Results

### 3.1 MG induces NET-associated responses that are masked by concurrent nuclease activity

**Fig. 1:**
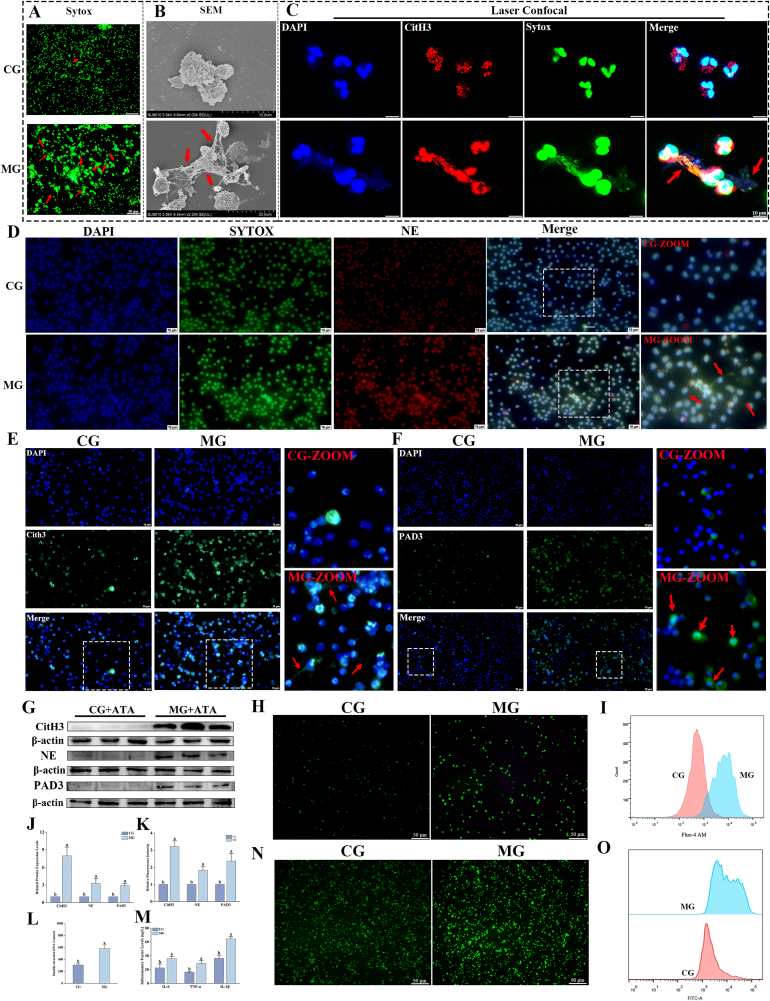
MG induces NET formation and immune activation in neutrophils. For NET visualization, neutrophils were pretreated with ATA (25 μM) before MG stimulation to inhibit MG-associated nuclease activity. (A) Representative Sytox Green staining of neutrophils stimulated with MG (80 MOI) (n = 3). Red arrows indicate NETs. (B) Representative scanning electron microscopy (SEM) images of NETs in MG-stimulated neutrophils (n = 3). Red arrows indicate NETs. (C) Representative confocal images of NETs induced by MG in neutrophils. Red arrows indicate NETs (n = 3). (D to F and K) Representative immunofluorescence images and quantification of neutrophil elastase (NE), citrullinated histone H3 (CitH3), and PAD3 fluorescence intensity in neutrophils after MG stimulation (n = 3). Red arrows in (D) and (E) indicate NETs; red arrows in (F) indicate nuclear translocation of PAD3. White dashed lines indicate enlarged regions. (G and J) Immunoblot analysis and quantification of CitH3, NE, and PAD3 protein levels (n = 6) in neutrophils after MG challenge. (H and I) Intracellular Ca^2+^ fluorescence intensity and representative flow cytometry plots in neutrophils after MG stimulation (n = 3). (L) dsDNA levels in culture supernatants after micrococcal nuclease digestion (n = 6). (M) ELISA quantification of inflammatory cytokine levels in neutrophils from the indicated groups (n = 6). (N-O) Intracellular ROS fluorescence intensity and representative flow cytometry plots in neutrophils after MG stimulation (n = 3). Data are presented as means ± SD from at least three independent experiments. Different lowercase letters indicate significant differences among groups (P < 0.05).

**Fig. 2:**
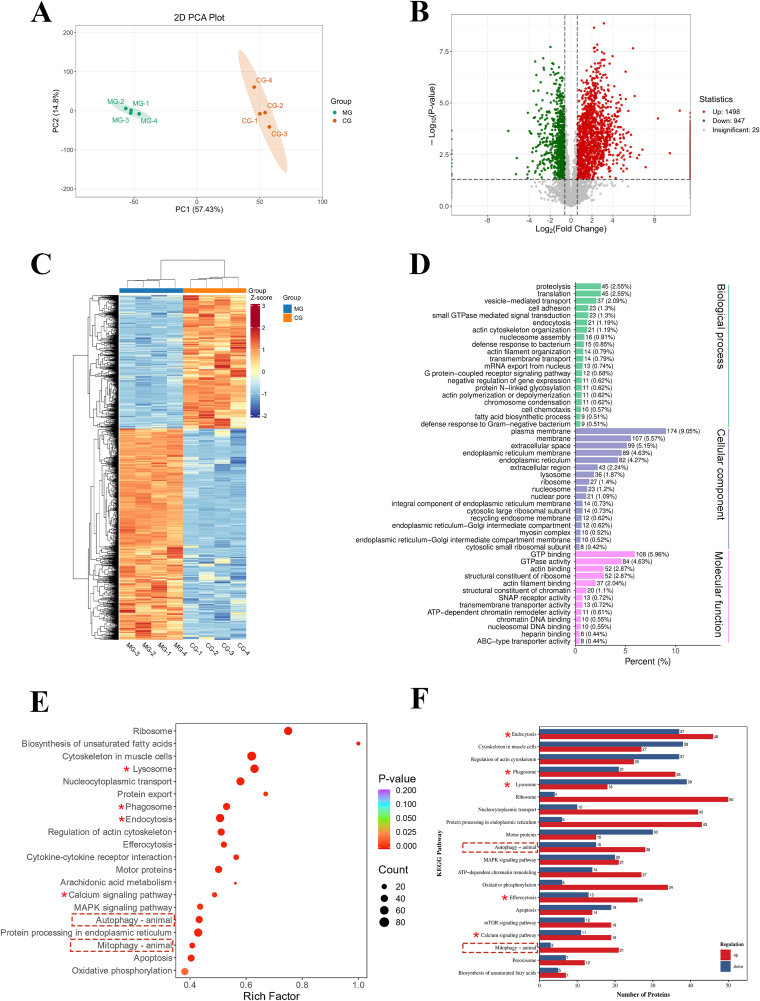
Proteomic analysis of neutrophils after MG stimulation (80 MOI, 1 h). (A) Principal component analysis (PCA) showing overall proteomic differences between the control group (CG) and MG group, as well as the degree of variation among samples within each group (n = 4). (B) Volcano plot showing the statistical significance and magnitude of differences in protein abundance between the CG and MG groups. (C) Hierarchical clustering heatmap of differentially expressed proteins between groups. (D) Gene Ontology (GO) enrichment analysis of the differentially expressed proteins. (E) Bubble plot of KEGG pathway enrichment analysis of the differentially expressed proteins. (F) Bar plot comparing KEGG pathway classifications of up- and down-regulated differentially expressed proteins. In (E) and (F), red asterisks indicate pathways shared between the two panels, and red boxes highlight the autophagy and mitophagy pathways.

We first asked whether MG could trigger NET-associated responses in chicken neutrophils and, if so, why obvious trap structures were difficult to visualize during direct MG challenge. To determine a suitable MG challenge dose, neutrophils were exposed to several MOIs, with phorbol 12-myristate 13-acetate (PMA) included as a positive control (S-Fig. 1 and S-Fig. 2). An MOI of 80 produced responses similar to those induced by PMA. However, in contrast to PMA-treated cells, extracellular DNA released after MG challenge was detected mainly in the culture supernatant, while obvious NET structures were not seen. This inconsistency suggested that MG might stimulate NET release while concurrently degrading the extracellular DNA scaffold through MG-associated nuclease activity, thereby obscuring direct trap visualization.

**Fig. 3:**
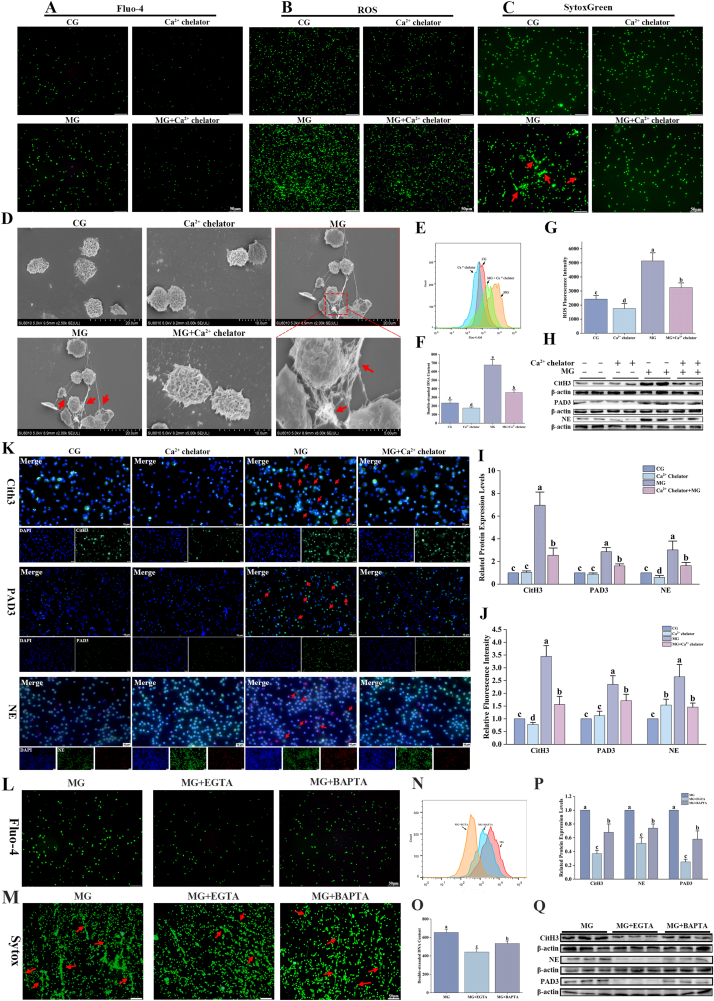
Extracellular Ca^2+^ influx is required for MG-induced oxidative signaling and NET release. To assess the contribution of Ca^2+^ signaling to MG-induced NET release, neutrophils were pretreated with the calcium chelators EGTA (10 μM, 1 min) and BAPTA (25 μM, 30 min) before MG challenge for 3 h. (A) Representative fluorescence microscopy images showing intracellular Ca^2+^ signals in neutrophils from the indicated groups, as detected by Fluo-4 staining (n = 3). (B) Representative fluorescence microscopy images showing intracellular ROS signals in neutrophils from the indicated groups (n = 3). (C) Representative Sytox Green staining images showing NET formation in the indicated groups. Red arrows indicate NETs (n = 3). (D) Representative SEM images showing NET release in the indicated groups. Red arrows indicate NETs, and red dashed boxes indicate enlarged regions in the MG group (n = 3). (E) Flow cytometric analysis of intracellular Ca^2+^ fluorescence intensity in neutrophils from the indicated groups (n = 3). (F) Extracellular dsDNA levels in the indicated groups (n = 6). (G) Quantification of intracellular ROS fluorescence intensity measured using a fluorescence microplate reader (n = 3). (H and I) Immunoblot analysis and quantification of NET-associated proteins (CitH3, PAD3, and NE) (n = 6) in the indicated groups (n = 3). (J to M) Representative immunofluorescence images and quantification of NET-associated proteins (CitH3, PAD3, and NE) in the indicated groups (n = 3). Red arrows indicate NETs or nuclear translocation of PAD3. To compare the relative contributions of extracellular and intracellular Ca^2+^ to MG-induced NET formation, extracellular or intracellular Ca^2+^ was chelated using EGTA or BAPTA, respectively. For extracellular Ca^2+^ chelation, the medium was pretreated with EGTA (10 μM) for 1 min before stimulation. For intracellular Ca^2+^ chelation, cells were pretreated with BAPTA (25 μM) for 30 min. (L) Representative fluorescence microscopy images showing intracellular Ca^2+^ signals detected by Fluo-4 staining (n = 3). (M) Representative Sytox Green staining images showing NET formation in the indicated groups. Red arrows indicate NETs. (N) Flow cytometric analysis of intracellular Ca^2+^ fluorescence intensity in the indicated groups (n = 3). (O) Extracellular dsDNA levels (n = 6). (P and Q) Immunoblot analysis (n = 3) and quantification of NET-associated proteins (CitH3, NE, and PAD3) (n = 6) in the indicated groups. Data are presented as means ± SD from at least three independent experiments. Different lowercase letters denote statistically significant differences among groups (P < 0.05).

In agreement with this possibility, MG showed strong nuclease activity in both the bacterial preparation and culture medium, whereas 25 μM ATA efficiently inhibited this activity without significantly changing extracellular dsDNA levels when used alone (S-Fig. 3). Under nuclease-inhibited conditions, MG-induced NET formation was readily detected by Sytox Green staining, SEM, immunofluorescence and WB. MG generated web-like extracellular structures resembling NETs (Fig. 1A–C), increased NET-associated markers, including citrullinated histone H3 (CitH3), neutrophil elastase (NE), and PAD3 (Fig. 1D–F and G–K), and raised extracellular dsDNA levels (Fig. 1L). Meanwhile, MG stimulation elevated intracellular Ca^2+^ levels in neutrophils (Fig. 1H and I), a key upstream event linked to NET formation, and increased intracellular ROS accumulation, as shown by ROS probe staining and flow-cytometric analysis (Fig. 1N and O). MG challenge also modified the expression of inflammatory mediators, including IL-1β, IL-6, and TNF-α (Fig. 1M). Collectively, these results indicate that MG activates a Ca^2+^/ROS-associated neutrophil response and promotes NET formation *in vitro*, although simultaneous MG-associated nuclease activity can hide evident trap accumulation.

### 3.2 Early proteomics identifies Ca^2+^, redox, and mitochondrial quality-control pathways during NET initiation

**Fig. 4:**
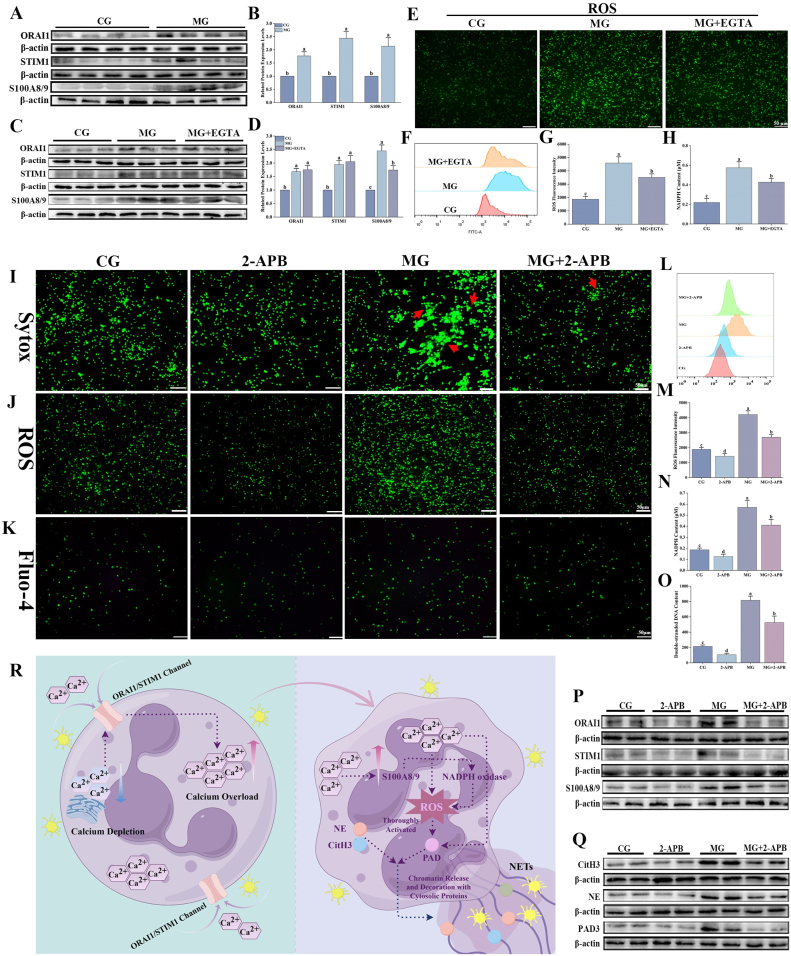
ORAI1/STIM1-associated Ca^2+^ entry links extracellular Ca^2+^ availability to oxidative signaling and MG-induced NET release. To investigate mechanisms potentially associated with MG-induced Ca^2+^ influx, neutrophils were challenged with MG (80 MOI) for 3 h before the indicated analyses. (A and B) Immunoblot analysis and quantification of ORAI1, STIM1, and S100A8/9 protein abundance (n = 6). To further assess the role of extracellular Ca^2+^ in MG-challenged neutrophils, cells were treated with EGTA (10 μM). (C and D) Immunoblot analysis of ORAI1/STIM1 and S100A8/9 protein expression in the indicated groups (n = 3). (E and F) Representative fluorescence microscopy (E) and flow cytometry (F) images showing intracellular ROS signals in the indicated groups (n = 3). (G) Quantification of intracellular ROS fluorescence intensity measured using a fluorescence microplate reader (n = 6). (H) NADPH-related levels in the indicated groups (n = 6). To assess the contribution of ORAI1/STIM1-associated Ca^2+^ entry to MG-induced NET release, neutrophils were pretreated with 2-APB (50 μM) for 1 h and then challenged with MG (80 MOI) for 3 h before the indicated analyses. (I) Representative Sytox Green staining images showing NET formation in the indicated groups (n = 3). (J) Representative fluorescence microscopy images showing intracellular ROS signals in the indicated groups (n = 3). (K) Representative fluorescence microscopy images showing intracellular Ca^2+^ signals detected by Fluo-4 staining in the indicated groups (n = 3). (L) Flow cytometric analysis of intracellular Ca^2+^ fluorescence intensity in the indicated groups (n = 3). (M) Quantification of intracellular ROS fluorescence intensity measured using a fluorescence microplate reader (n = 6). (N) NADPH-related levels in the indicated groups (n = 6). (O) Extracellular dsDNA levels (n = 6). (P to Q) Immunoblot analysis of NET-associated proteins (CitH3, NE, and PAD3), ORAI1/STIM1-associated proteins, and S100A8/9 in the indicated groups (n = 6). (R) Schematic model of MG-induced NET release mediated by extracellular Ca^2+^ influx. The schematic was created with Figdraw (ID: PWOIUe1ea3). Data are presented as means ± SD from at least three independent experiments. Different lowercase letters denote statistically significant differences among groups (P < 0.05).

After establishing that MG can induce NET formation while concealing extracellular trap accumulation, we next aimed to identify the intracellular events that initiate this response. NET release was examined at several time points after MG exposure (S-Fig. 4). Because NETs were not easily detected at 1 h, whereas marked release was observed at 2 and 3 h, the 1 h time point was chosen to capture early signaling before clear NET execution. Proteomic profiling of neutrophils exposed to MG (80 MOI) for 1 h detected 2445 differentially regulated proteins, including 1498 up-regulated and 947 down-regulated proteins (Fig. 2B). GO enrichment analysis showed that MG challenge rapidly induced broad cellular responses in neutrophils. More notably, KEGG pathway enrichment analysis showed significant enrichment of calcium signaling, autophagy, ROS-related responses, mitophagy, lysosome, phagosome, and MAPK-associated pathways (Fig. 2E and F). These early alterations suggested that MG-triggered NET initiation is not a single terminal event, but instead represents a coordinated remodeling process involving signaling, organelle quality control, and membrane-trafficking programs.

**Fig. 5:**
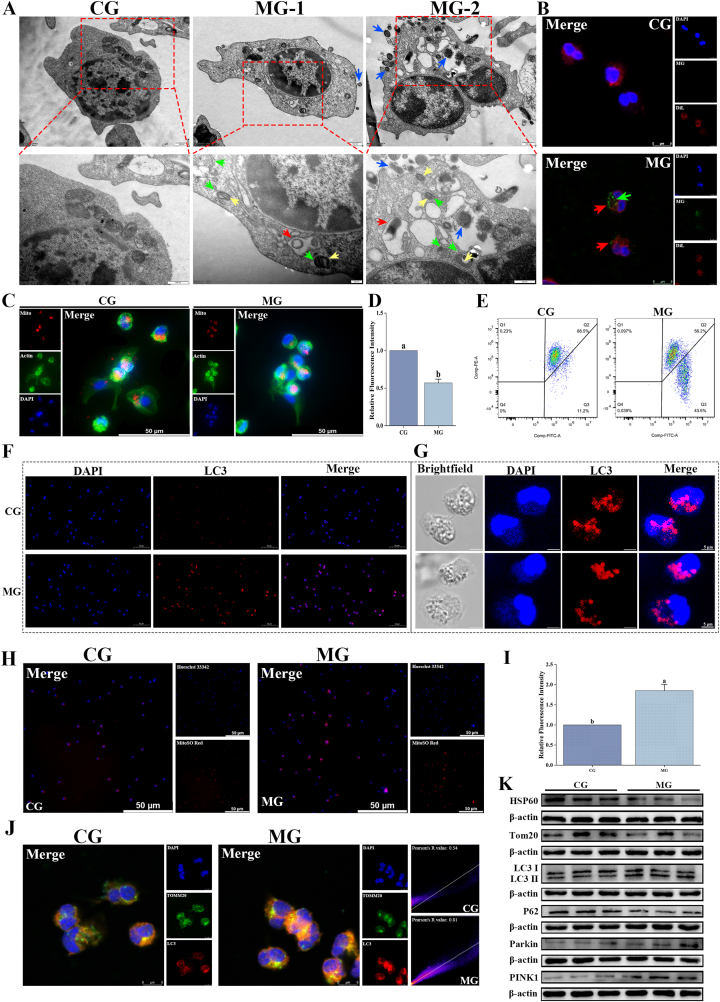
MG induces early mitophagy-associated responses in neutrophils. Neutrophils were exposed to MG (80 MOI) for 1 h and then subjected to the indicated analyses. (A) Representative TEM images of neutrophils (n = 3). Yellow arrows indicate mitochondria, blue arrows indicate putative MG, green arrows indicate mitophagy-related structures, and red arrows indicate autolysosomes. (B) Representative confocal images showing the subcellular localization of MG after incubation with neutrophils (n = 3). Red arrows indicate MG associated with the neutrophil surface, and green arrows indicate intracellular MG. (C and D) Representative confocal images and quantification of mitochondrial fluorescence in neutrophils from the control group (CG) and MG group (n = 3). (E) Flow cytometric analysis of mitochondrial membrane potential in neutrophils from the CG and MG groups using the JC-1 fluorescent probe (n = 3). (F) Representative immunofluorescence images showing LC3 fluorescence intensity in neutrophils from the CG and MG groups (n = 3). (G) Representative confocal images showing LC3 staining patterns in neutrophils from the CG and MG groups (n = 3). (H and I) Representative fluorescence microscopy images showing mitochondrial ROS signals labeled with MitoSOX™ Red in the indicated groups (n = 3). (J) Representative confocal images showing the localization of TOMM20 and LC3 in neutrophils from the CG and MG groups (n = 3). (K) Western blot analysis of mitophagy-related proteins in neutrophils from the CG and MG groups (n = 6). Data are presented as means ± SD from at least three independent experiments. Different lowercase letters indicate significant differences among groups (P < 0.05).

To define the protein composition of MG-induced NETs, NET fractions isolated from neutrophils stimulated with MG (80 MOI) for 3 h were analyzed by mass spectrometry (S-Fig. 5). In total, 610 proteins were identified, including proteins overlapping with or related to previously reported NET-associated proteomes [[48], [49], [50], [51]]. Functional annotation showed enrichment in pathways associated with phagosome, regulation of actin cytoskeleton, autophagy/mitophagy, calcium signaling, and MAPK signaling, with proteins originating mainly from the nucleus, cytoplasm, and membrane-associated compartments. Together, these findings support the interpretation that MG-induced extracellular material is compositionally consistent with NET-associated release and identify calcium signaling, autophagy/mitophagy, and MAPK-associated programs as possible upstream modules in MG-triggered NET initiation.

### 3.3 Extracellular Ca^2+^ influx sustains ROS/NADPH-associated oxidative activation

Because MG challenge elevated intracellular Ca^2+^ and ROS levels, and calcium signaling pathways were enriched in the early proteomic analysis, we next investigated whether the classical Ca^2+^/ROS signaling axis was involved in MG-induced NET formation. MG stimulation strongly increased intracellular Ca^2+^ levels in neutrophils, as indicated by Fluo-4 fluorescence imaging and flow-cytometric analysis (Fig. 3A and E), and also promoted intracellular ROS accumulation (Fig. 3B and G). In contrast, Ca^2+^ chelation markedly decreased both Ca^2+^ signals and ROS accumulation in MG-challenged neutrophils. Functionally, Ca^2+^ depletion strongly inhibited MG-induced NET release, as shown by fewer Sytox Green positive extracellular structures and reduced NET-like structures under SEM (Fig. 3C and D). Similarly, Ca^2+^ chelation lowered extracellular dsDNA levels (Fig. 3F) and reduced the abundance of NET-associated proteins, including CitH3, PAD3, and NE (Fig. 3H–K). These results indicate that Ca^2+^ signaling participates in oxidative activation and NET execution in MG-stimulated neutrophils.

We then applied this MG-induced NET model to compare the relative effects of extracellular and intracellular Ca^2+^ chelation. Both EGTA, which chelates extracellular Ca^2+^, and BAPTA, which chelates intracellular Ca^2+^, decreased intracellular Ca^2+^ signals compared with MG treatment alone; however, the inhibitory response was stronger in the EGTA-treated group (Fig. 3L and N). Consistent with this trend, EGTA showed a greater suppressive effect than BAPTA on extracellular dsDNA release and NET-associated proteins protein expression (Fig. 3M and O–Q). These findings suggest that extracellular Ca^2+^ influx has an important role in maintaining the Ca^2+^/ROS-associated signaling cascade that promotes MG-induced NET formation.

To further investigate how MG challenge enhances extracellular Ca^2+^ influx, we focused on the ORAI1/STIM1 pathway, a canonical store-operated Ca^2+^ entry module previously identified in our work as being activated during MG infection [52]. Compared with the control group, MG stimulation strongly increased ORAI1 and STIM1 abundance, supporting activation of this Ca^2+^ entry-associated signaling module in MG-challenged neutrophils. In parallel, MG also levated S100A8/9 increased the expression, a Ca^2+^-binding inflammatory protein complex associated with neutrophil activation and inflammatory amplification (Fig. 4A and B). We then examined whether extracellular Ca^2+^ availability affected this signaling response. Compared with MG treatment alone, EGTA showed limited effects on ORAI1 and STIM1 protein abundance; however, densitometric quantification indicated that EGTA significantly decreased MG-induced S100A8/9 expression (Fig. 4C and D). Likewise, EGTA markedly reduced NADPH-related activity and intracellular ROS accumulation in MG-challenged neutrophils (Fig. 4E–H). These results suggest that MG activates an ORAI1/STIM1-associated Ca^2+^ entry response, and that extracellular Ca^2+^ availability is closely related to S100A8/9 induction and ROS/NADPH-associated oxidative activation during MG-induced neutrophil responses.

To further evaluate the functional contribution of ORAI1/STIM1-associated Ca^2+^ entry to MG-induced neutrophil activation, neutrophils were pretreated with 2-APB, an inhibitor commonly applied to block ORAI1/STIM1-associated Ca^2+^ entry, before MG challenge. Compared with MG treatment alone, 2-APB greatly decreased intracellular Ca^2+^ signals (Fig. 4K and L). Furthermore, 2-APB reduced ROS accumulation, NADPH-related activity, S100A8/9 abundance, and NET release in MG-challenged neutrophils (Fig. 4I–P). Overall, these data indicate that ORAI1/STIM1-associated extracellular Ca^2+^ entry contributes to the Ca^2+^/ROS-associated signaling cascade involved in MG-induced NET formation.

### 3.4 Mitochondrial dysfunction and mitophagy-associated remodeling link oxidative activation to PAD-associated NET execution

After confirming the involvement of Ca^2+^/ROS signaling in MG-induced NET formation, we next examined which downstream events might occur following the increase in intracellular ROS, with particular focus on mitochondrial dysfunction and mitophagy-associated responses suggested by the proteomic analysis. To detect early mitochondrial alterations before robust NET release, neutrophils were collected 1 h after MG challenge (80 MOI) and subjected to ultrastructural and molecular analyses. TEM showed autophagosome-like and mitophagy-related structures in MG-treated neutrophils, together with MG organisms at the cell surface and inside intracellular compartments (Fig. 5A and B). MG challenge also clearly reduced mitochondrial fluorescence intensity (Fig. 5C–D), disturbed mitochondrial membrane potential (Fig. 5E), and increased mitochondrial ROS accumulation, as shown by MitoSOX™ Red staining (Fig. 5H and I). MG stimulation further strengthened LC3 signals and promoted LC3-related cytoplasmic accumulation (Fig. 5F and G), indicating activation of autophagy/mitophagy-related remodeling in neutrophils. Consistent with these results, confocal colocalization analysis of TOMM20 and LC3 showed increased TOMM20–LC3 colocalization after MG infection (Fig. 5J). Western blot analysis further supported these imaging results: MG challenge reduced the levels of the mitochondrial proteins HSP60 and TOMM20, decreased P62 expression, and increased LC3 and the mitophagy-initiation-related proteins PINK1 and Parkin (Fig. 5K). Taken together, these findings indicate that MG infection induces early mitochondrial dysfunction, mitochondrial ROS accumulation, and autophagy/mitophagy-related remodeling in neutrophils before marked NET release.

**Fig. 6:**
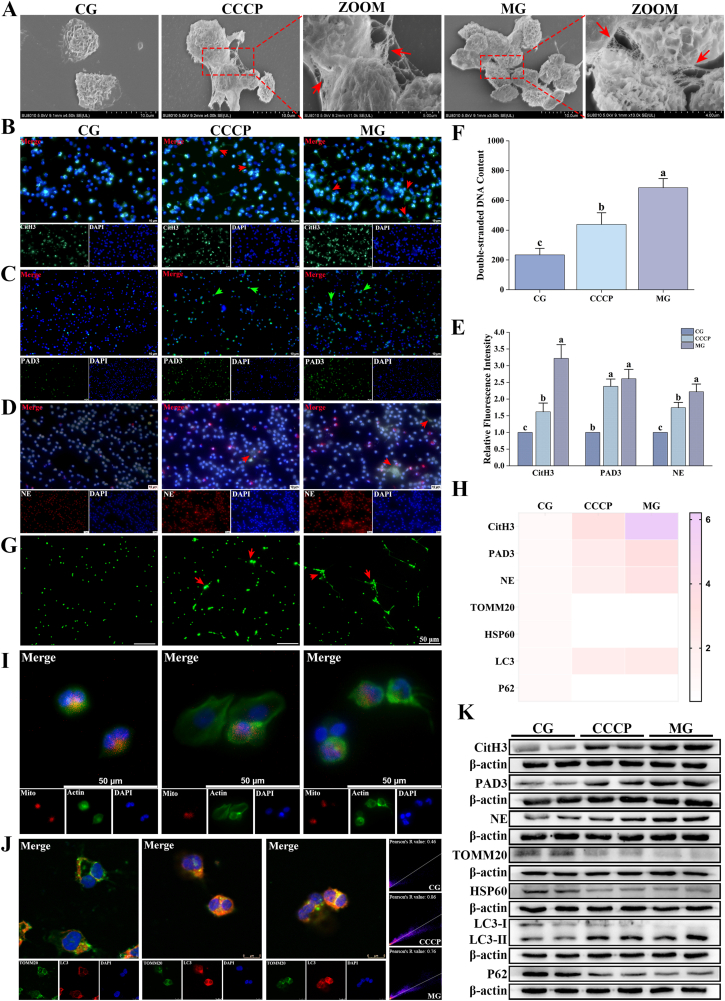
Mitophagy activation promotes NET formation in neutrophils. Neutrophils were treated with carbonyl cyanide m-chlorophenyl hydrazone (CCCP, 10 μM) for 2 h or stimulated with MG (80 MOI) for 3 h. (A) Representative SEM images showing NETs in neutrophils after CCCP or MG treatment. Red dashed boxes indicate enlarged regions, and red arrows indicate NETs (n = 3). (B to D and E) Representative immunofluorescence images and quantification of NE, CitH3, and PAD3 fluorescence intensity in neutrophils after CCCP or MG stimulation (n = 3). Red arrows in (B) and (D) indicate NETs, and green arrows in (C) indicate nuclear translocation of PAD3. (E) Quantification of immunofluorescence signals (n = 3). (F) Extracellular dsDNA levels (n = 6). (I) Representative confocal images showing mitochondrial fluorescence intensity (n = 3). (J) Representative confocal images showing the localization of TOMM20 and LC3 (n = 3). (H and K) Western blot analysis of NET- and mitophagy-related proteins (n = 6). Data are presented as means ± SD from at least three independent experiments. Different lowercase letters indicate significant differences among groups (P < 0.05).

Although oxidative stress has been associated with mitochondrial quality-control responses, the link between mitochondrial ROS and MG-induced mitophagy-related remodeling in neutrophils was still unclear. Therefore, Mito-TEMPO, a mitochondria-targeted superoxide scavenger, was used to determine whether mitochondrial ROS contributes to mitochondrial changes caused by MG challenge. As expected, Mito-TEMPO reduced MG-induced mitochondrial ROS accumulation and reduced total intracellular ROS levels (S-Fig. 6B–E). Mito-TEMPO treatment also partly recovered mitochondrial fluorescence intensity in MG-challenged neutrophils (S-Fig. 6A). Moreover, TEM showed that Mito-TEMPO relieved MG-induced mitochondrial injury and reduced autophagy/mitophagy-like ultrastructural changes (S-Fig. 6F). These results suggest that mitochondrial ROS contributes to MG-induced mitochondrial damage and mitophagy-related remodeling in neutrophils.

To determine whether mitophagy-associated activation could enhance NET release, neutrophils were exposed to the mitophagy activator carbonyl cyanide m-chlorophenyl hydrazone (CCCP) [53]. CCCP generated web-like extracellular structures similar to NETs, elevated extracellular dsDNA levels, and promoted extracellular accumulation of CitH3 and NE, together with stronger nuclear PAD3 signals (Fig. 6A–F). Meanwhile, CCCP decreased mitochondrial fluorescence intensity and enhanced TOMM20–LC3 colocalization, accompanied by parallel alterations in mitophagy-related proteins (Fig. 6H–K). These findings support the view that mitophagy-associated activation can strengthen NET-associated responses.

**Fig. 7:**
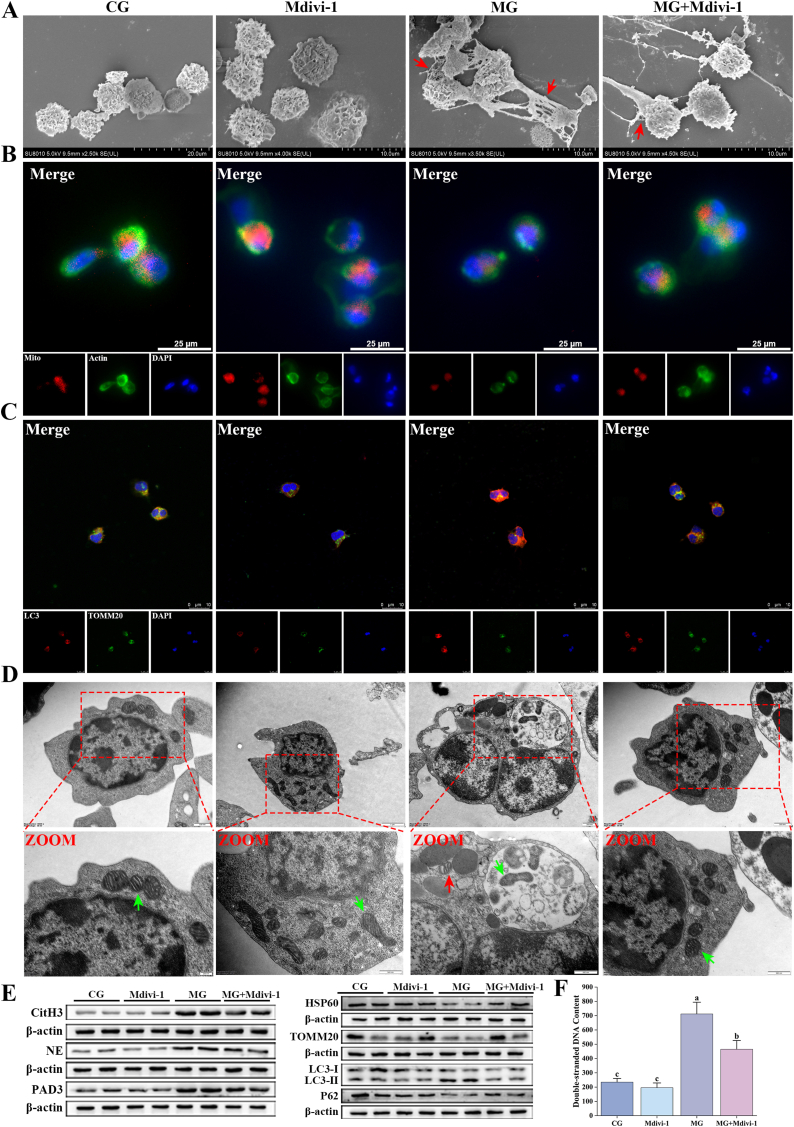
Pharmacological inhibition of mitophagy attenuates MG-induced NET formation. Neutrophils were pretreated with Mdivi-1 (20 μM) for 2 h and then challenged with MG (80 MOI) for 3 h. (A) Representative SEM images showing NETs in neutrophils from the indicated groups (n = 3). Red arrows indicate NETs. (B) Mitochondrial fluorescence intensity in neutrophils from the indicated groups, with representative confocal images shown (n = 3). (C) Representative confocal images showing the localization of TOMM20 and LC3 in neutrophils from the indicated groups (n = 3). (D) Representative TEM images showing ultrastructural changes in neutrophils after the indicated treatments (n = 3). Red dashed boxes indicate enlarged regions. (E) Western blot analysis of NET and mitophagy-related proteins (n = 6). (F) Extracellular dsDNA levels (n = 6). Data are presented as means ± SD from at least three independent experiments. Different lowercase letters indicate significant differences among groups (P < 0.05).

We then asked whether this mitophagy-associated module also participates in MG-induced NET formation. During MG challenge, neutrophils were exposed to Mdivi-1, a mitophagy inhibitor [54]. Mdivi-1 alone failed to induce NET formation and did not completely prevent MG-triggered NET release, but it clearly reduced the intensity of this response (Fig. 7A). Compared with MG treatment alone, MG plus Mdivi-1 enhanced mitochondrial fluorescence intensity, recovered TOMM20 signals, lowered LC3 fluorescence, and reduced mitophagy-related ultrastructural alterations (Fig. 7B–D). Functionally, Mdivi-1 markedly decreased extracellular dsDNA release and weakened MG-induced increases in CitH3, PAD3, and NE, while partly reversing changes in mitophagy-related proteins (Fig. 7E and F). These results indicate that mitophagy-associated regulation participates in MG-induced NET formation, although it is not the only mechanism driving this process.

**Fig. 8:**
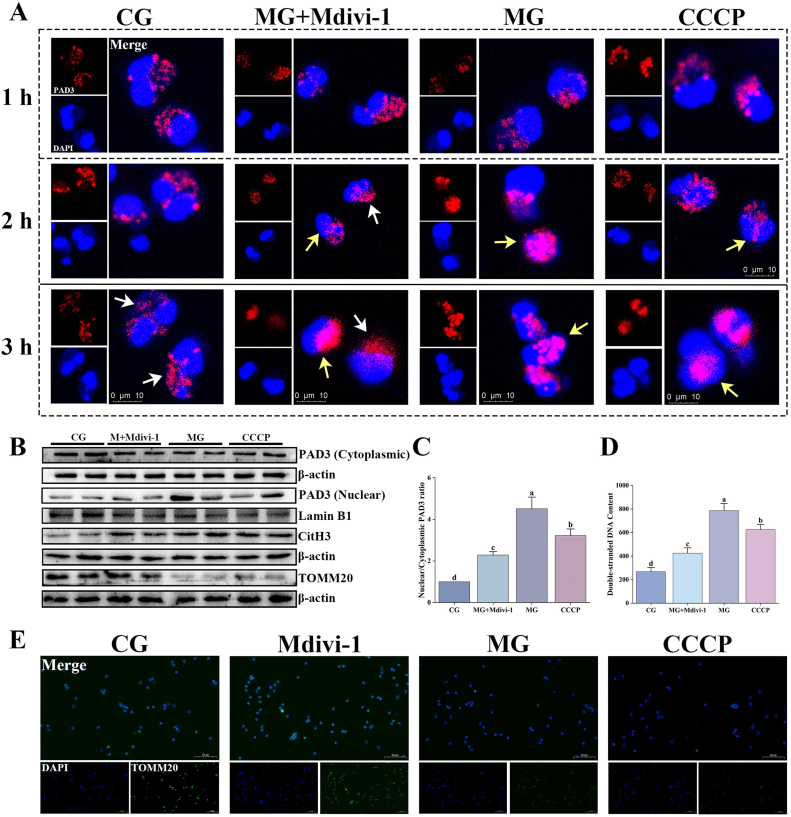
Mitophagy-associated regulation promotes PAD3 nuclear redistribution during MG-induced NET formation. To investigate the potential mechanism by which mitophagy-associated regulation contributes to NET release, neutrophils were divided into the CG, MG + Mdivi-1, MG, and CCCP groups. Because CCCP treatment was initiated at a different reference point from MG stimulation, the first time point shown for the CCCP group represents the baseline state before CCCP exposure. (A) Representative confocal images showing the time-dependent subcellular distribution of PAD3 in the indicated groups. White arrows indicate cytoplasmic PAD3, and yellow arrows indicate nuclear PAD3 (n = 3). (B–C) Western blot analysis and quantification of PAD3 distribution in nuclear and cytoplasmic fractions, together with CitH3 and TOMM20 protein levels in the indicated groups (n = 6). (D) Extracellular dsDNA levels in the indicated groups (n = 6). (E) Representative immunofluorescence images and quantification of TOMM20 fluorescence intensity in the indicated groups (n = 3). Data are presented as means ± SD from at least three independent experiments. Different lowercase letters indicate significant differences among groups (P < 0.05).

To assess PAD-family-associated chromatin execution, PAD3 was selected as the main focus. Preliminary screening indicated that both PAD2 and PAD3 transcripts were increased after MG stimulation, whereas PAD3 showed a more consistent protein signal in this experimental system and underwent nuclear redistribution during MG-induced NET formation. Therefore, PAD3 was used as the principal PAD-family-associated readout in this study. To examine how mitophagy-associated regulation supports NET release, PAD3 redistribution was analyzed using the mitophagy activator CCCP and the inhibitor Mdivi-1. At 1 h, PAD3 was mainly distributed in the cytoplasm. By 2 h, nuclear redistribution of PAD3 was evident in the MG + Mdivi-1, MG, and CCCP groups, and by 3 h this redistribution was further strengthened in the MG and CCCP groups (Fig. 8A). Consistently, nuclear/cytoplasmic fractionation followed by Western blotting demonstrated that the nuclear-to-cytoplasmic PAD3 ratio was markedly lower in the MG + Mdivi-1 group than in the MG group. This change was accompanied by reduced CitH3 abundance (Fig. 8B–C), increased TOMM20 levels and fluorescence intensity (Fig. 8E), and decreased extracellular dsDNA release (Fig. 8D). These findings suggest that mitophagy-associated regulation supports MG-induced NET release by facilitating PAD3 nuclear redistribution and PAD-dependent chromatin execution.

**Fig. 9:**
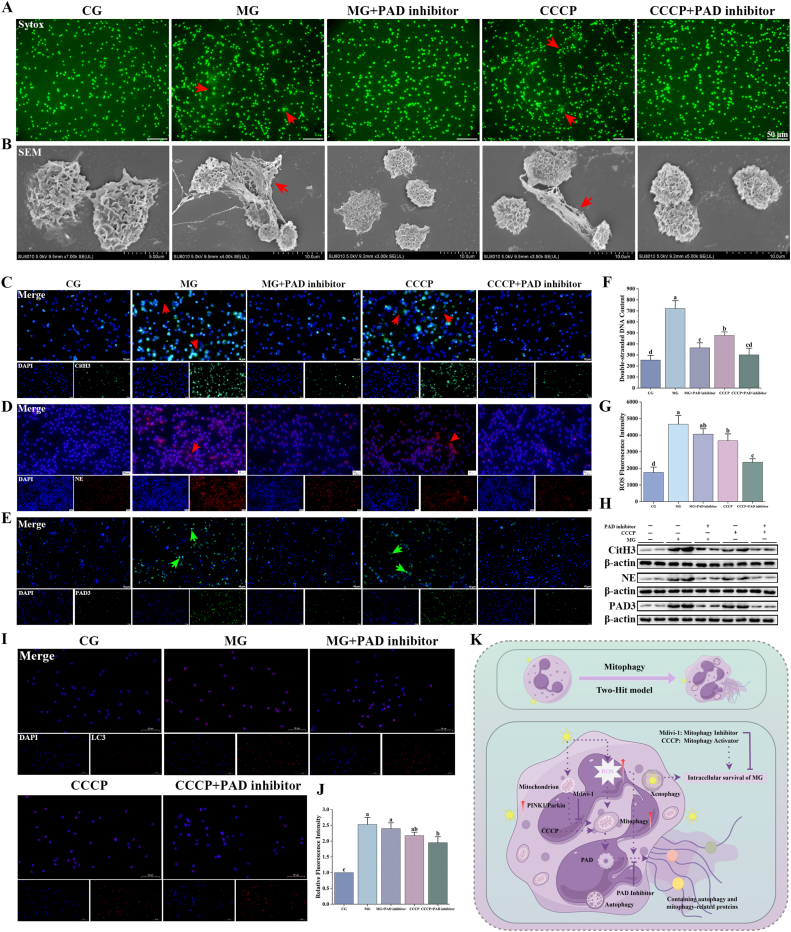
Mitophagy-associated remodeling contributes to PAD-dependent NET execution. To examine the relationship between mitophagy and PAD signaling, neutrophils were pretreated with the PAD inhibitor Cl-amidine (5 μM) for 30 min and then stimulated with CCCP (10 μM, 2 h) or MG (80 MOI, 3 h). (A) Representative Sytox Green staining images showing NETs in the indicated groups. Red arrows indicate NETs (n = 3). (B) Representative SEM images showing NET release in the indicated groups. Red arrows indicate NETs (n = 3). (C to E) Representative immunofluorescence images showing the fluorescence intensity and localization of NET-associated proteins (n = 3), including CitH3, PAD3, and NE. Red arrows in (C) and (D) indicate NETs, and green arrows in (E) indicate nuclear translocation of PAD proteins. (F) Extracellular dsDNA levels (n = 6). (G) ROS fluorescence intensity measured using a fluorescence microplate reader (n = 6). (H) Western blot analysis of NET-associated proteins (CitH3, PAD3, and NE) in the indicated groups (n = 6). (I and J) Representative immunofluorescence images and quantification of LC3 fluorescence intensity in neutrophils from the indicated groups (n = 3). (K) Schematic model illustrating MG-induced NET formation through the mitophagy/PAD pathway. The schematic was created with Figdraw (ID: IRSIAfd8ff). Data are presented as means ± SD from at least three independent experiments. Different lowercase letters indicate significant differences among groups (P < 0.05).

Given that PAD enzymes act as central effectors of NET formation, we next asked whether mitophagy-associated regulation could drive NET release independently or instead depended on PAD activation. To address this issue, neutrophils were treated with the mitophagy activator CCCP or MG in the presence or absence of a PAD inhibitor. PAD inhibition blocked NET release induced by either CCCP or MG, as shown by Sytox Green staining (Fig. 9A), SEM (Fig. 9B), extracellular dsDNA measurements (Fig. 9F), and reduced ROS, CitH3, NE, and PAD3 expression (Fig. 9C–E and G-H). In contrast, PAD inhibition produced only minor effects on LC3 fluorescence intensity (Fig. 9I). These results indicate that mitophagy-associated regulation does not function as an independent terminal pathway, but instead converges with PAD-dependent chromatin execution.

**Fig. 10:**
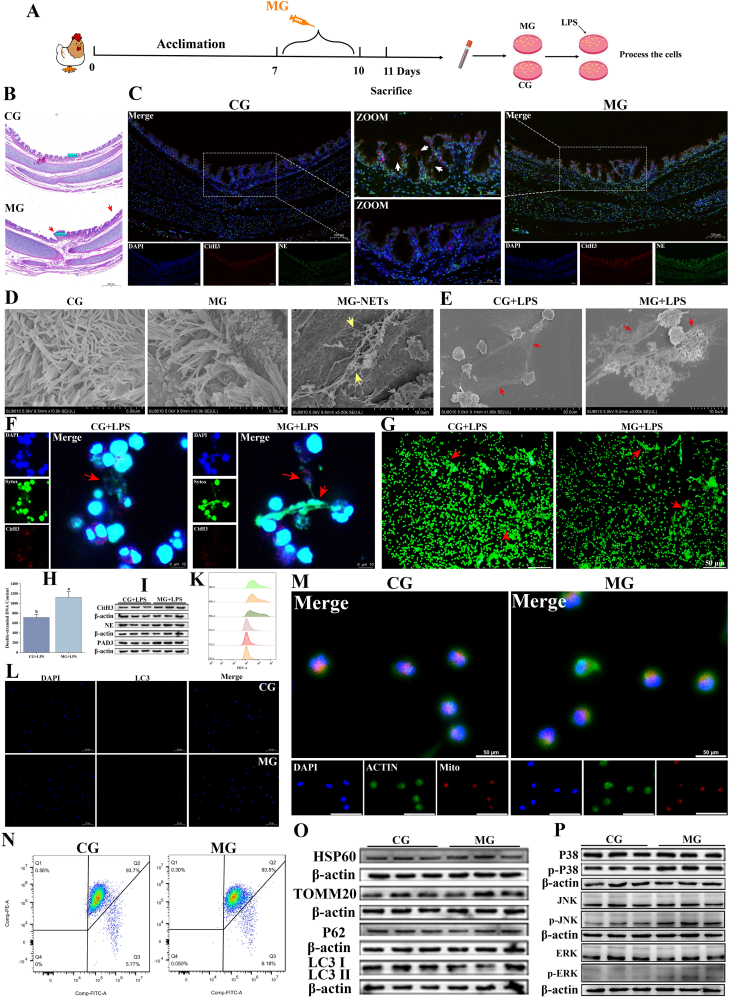
MG infection alters neutrophil-associated responses *in vivo*. A chick infection model was established by air sac injection of 0.2 mL MG suspension (1 × 10^^9^ CCU/mL), together with auxiliary intranasal inoculation. On day 4 after infection, blood was collected from the control group (CG) and MG group, and neutrophils were isolated for the indicated analyses (n = 15). (A) Schematic overview of the experimental design. (B) Representative hematoxylin and eosin (H&E) staining of tracheal sections from the indicated groups (n = 3). Red arrows indicate epithelial detachment and disruption of mucosal integrity. (C) Representative immunofluorescence images showing NET-associated signals in tracheal tissue, labeled with CitH3 and NE (n = 3). White arrows indicate overlapping CitH3 and NE fluorescence signals. (D) Representative SEM images showing ciliary damage in tracheal tissue from the indicated groups (n = 3). Yellow arrows indicate extracellular structures morphologically consistent with NET-like material. (E) Representative SEM images showing NET release from neutrophils isolated from the CG and MG groups after LPS stimulation (5 μg/mL, 3 h) (n = 3). Red arrows indicate NETs. (F) Representative confocal images showing NETs released from neutrophils isolated from the CG and MG groups after LPS stimulation, labeled with Sytox Green and anti-CitH3 antibody (n = 3). Red arrows indicate NETs. (G) Representative fluorescence microscopy images of Sytox Green–stained NETs in the indicated groups. (H) Extracellular dsDNA levels in the indicated groups (n = 6). (I) Immunoblot analysis of NET-associated proteins (CitH3, NE, and PAD3) in the indicated groups (n = 3). (K) Flow cytometric analysis of ROS fluorescence intensity in neutrophils isolated from the CG and MG groups (n = 3). (L) Immunofluorescence analysis of LC3 fluorescence intensity in neutrophils isolated from the indicated groups (n = 3). (M) Representative confocal images showing mitochondrial fluorescence intensity in neutrophils from the indicated groups labeled with a mitochondrial probe (n = 3). (N) Flow cytometric analysis of mitochondrial membrane potential in neutrophils from the indicated groups using the JC-1 probe (n = 3). (O and P) Immunoblot analysis of mitophagy-related proteins (HSP60, TOMM20, P62, and LC3) and MAPK pathway–related proteins in neutrophils isolated from the indicated groups (n = 3). Data are presented as means ± SD from at least three independent experiments. Different lowercase letters indicate significant differences among groups (P < 0.05).

**Fig. 11:**
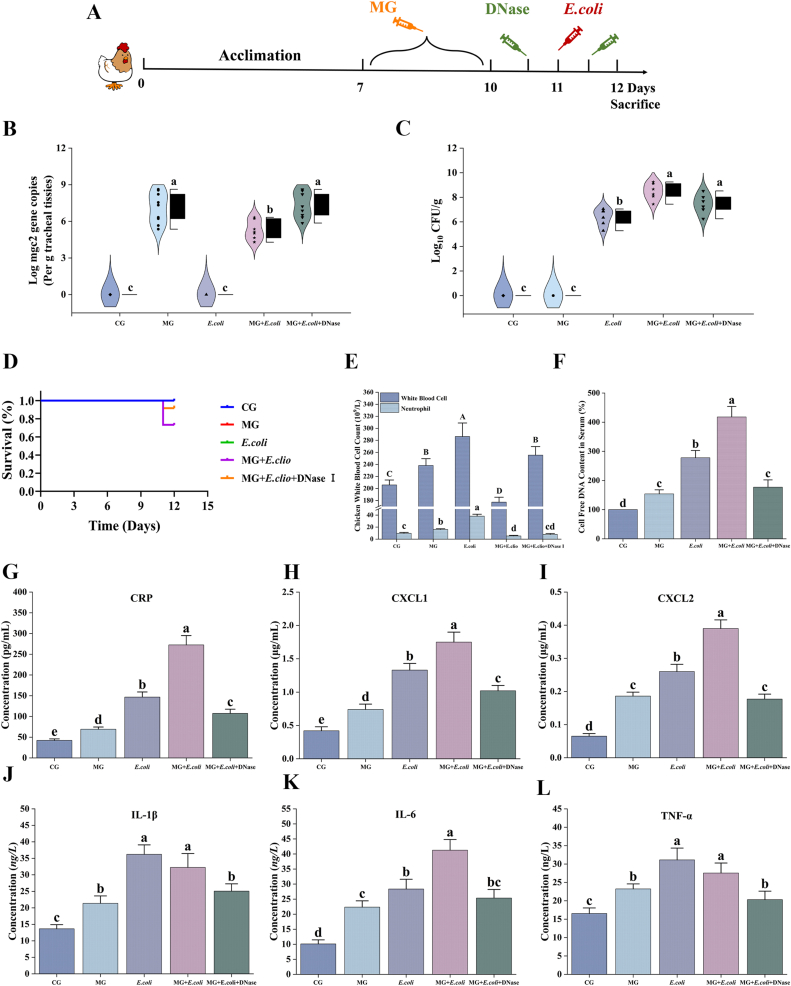
MG and *E. coli* co-infection exacerbates disease severity and is associated with enhanced NET release. To determine whether MG-induced changes in host neutrophil responses contribute to the severity of co-infection, a chick co-infection model was established (n = 15). Chicks were first infected with MG by air sac injection of 0.2 mL MG suspension (1 × 10^^9^ CCU/mL) combined with intranasal inoculation, followed by intraperitoneal injection of 0.2 mL E*. coli* JD37 suspension (2 × 10^^9^ CFU/mL). DNase I (50 U/chick) was administered intraperitoneally 12 h before and 12 h after *E. coli* JD37 challenge. (A) Experimental design. (B) Tracheal MG colonization in the indicated groups (n = 9). (C) Intestinal *E. coli* JD37 colonization in the indicated groups (n = 6). (D) Survival curves of the indicated groups. (E) Hematological parameters in the indicated groups (n = 6). (F) Serum dsDNA levels in the indicated groups (n = 6). (G) Serum C-reactive protein levels in the indicated groups (n = 6). (H and I) Serum chemokine levels in the indicated groups (n = 6). (J to L) Serum levels of inflammatory cytokines, including IL-1β, IL-6, and TNF-α, in the indicated groups (n = 6). Data are presented as means ± SD from at least three independent experiments. Different lowercase letters indicate significant differences among groups (P < 0.05).

### 3.5 Pathogen nuclease activity rewires extracellular DNA fate and dampens NET-driven inflammatory amplification

Since mycoplasmas have undergone extensive reductive evolution and consequently lack many biosynthetic pathways needed for de novo synthesis of essential metabolites, including nucleotides, we hypothesized that MG may use extracellular trap degradation not only to weaken antimicrobial trap integrity at the local host pathogen interface, but also to modify the downstream biological accessibility of trap-derived materials. Since prolonged and efficient labeling of neutrophil extracellular DNA was technically difficult HD11 macrophages were used as a surrogate system for tracing extracellular DNA.

Proliferating HD11 cells were labeled with EdU before PMA stimulation. PMA strongly induced macrophage extracellular trap (MET) formation in HD11 cells (S-Fig. 7A). EdU-positive extracellular MET structures were easily detected after PMA stimulation but were largely lost after co-incubation with MG; in contrast, EdU-labeled METs reappeared when the nuclease inhibitor ATA was present, consistent with MG-associated nuclease activity-mediated MET degradation (S-Fig. 7B). When PMA-induced METs were isolated and co-incubated with MG, EdU fluorescence was observed in DAPI-labeled MG, and this signal decreased in the presence of EDTA, suggesting that nuclease-dependent degradation supports the MG access to extracellular trap-derived DNA signals/materials (S-Fig. 7C).

The consequences of trap degradation are not restricted to pathogen access to nucleic acid products; degradation may also shape the extent to which extracellular traps stimulate neighboring host cells. Therefore, we examined whether intact NETs retained immunostimulatory activity in recipient macrophages and epithelial cells. In HD11 cells, intact NETs promoted inflammatory activation and favored an M1-like phenotype, whereas in CETs they enhanced inflammatory injury and barrier-associated damage (S-Figs. 8–12). These effects became more evident when MG nuclease activity was inhibited, indicating that MG-mediated trap degradation reduces NET-dependent immune amplification. Together, these findings indicate that MG not only escapes extracellular traps, but also actively reshapes their fate and downstream immunological effects.

### 3.6 MG infection primes systemic neutrophil redox responsiveness and aggravates NET-associated pathology during secondary challenge

Beyond inducing NET formation, MG could degrade extracellular traps, access trap-derived degradation products, and modify downstream host immune responses. These observations suggest that, within the local immune microenvironment, MG may regulate NET biology in a manner that restricts excessive NET-dependent immune amplification and may be compatible with persistent colonization. Although the direct contribution of trap-derived products to MG growth or dissemination remains to be determined. We reasoned that if MG reshapes NET biology at the mucosal interface, infection may also modify systemic neutrophil responsiveness and affect disease severity during a subsequent infectious challenge. Therefore, we next asked how MG infection affects neutrophil behavior and NET-associated responses *in vivo*.

To determine how MG infection modifies neutrophil-related responses *in vivo*, a chick model of MG infection associated with tracheal Th1 immune imbalance was established (Fig. 10A) [31,55]. MG infection impaired tracheal mucosal integrity and increased mucosal thickness (Fig. 10B). SEM further showed decreased ciliary abundance, damaged ciliary structure, and extracellular structures morphologically consistent with NET-like material in the MG group (Fig. 10D). Similarly, immunofluorescence staining revealed stronger CitH3 and NE signals in tracheal tissues from MG-infected chicks, suggesting local NET-associated responses at the main colonization site (Fig. 10C).

We then examined whether this local mucosal response was accompanied by systemic neutrophil priming. Circulating neutrophils were isolated from control and MG-infected chicks and subsequently challenged *ex vivo* with LPS. Neutrophils from both groups were able to release NETs after LPS exposure, although the morphology of LPS-induced NETs differed from that observed after direct MG stimulation *in vitro* (Fig. 10E–G). Notably, compared with the CG + LPS group, the MG + LPS group displayed higher extracellular dsDNA levels and increased NET-associated protein abundance, indicating that previous MG infection primes neutrophils for stronger NET release after subsequent stimulation (Fig. 10H and I). We then further evaluated whether the NET-related responses observed *in vivo* resembled those caused by direct MG stimulation *in vitro*. The results showed that, although neutrophils from the MG group exhibited increased ROS signals, no significant differences were observed in LC3 fluorescence intensity, mitochondrial abundance, mitochondrial membrane potential damage, or mitophagy-related protein levels (Fig. 10K–O). Based on the *in vitro* proteomic data and known signaling pathways involved in NET formation, MAPK-associated signaling was then examined, and MAPK-related protein levels were found to be significantly elevated in neutrophils isolated from MG-infected chicks (Fig. 10P). These data suggest that direct MG stimulation *in vitro* was associated with mitophagy-linked NET regulation, whereas circulating neutrophil priming during the tracheal Th1 immune-imbalance phase *in vivo* appeared to be mediated mainly by ROS/MAPK-associated inflammatory signaling.

Although single MG infection showed altered neutrophil responsiveness, it was not sufficient to determine whether this reprogramming could result in aggravated pathology during a later infectious challenge. Therefore, an *in vivo* co-infection model involving MG and *E. coli* JD37 was established, and DNase I was administered to degrade extracellular DNA/NET-associated material, thereby assessing the pathological relevance of MG-induced neutrophil reprogramming during secondary infection (Fig. 11A; S-Fig. 13). Prior MG infection promoted *E. coli* colonization in the chick intestine, whereas co-infection was associated with decreased MG colonization in the trachea (Fig. 11B–C). Although DNase I treatment did not significantly change pathogen burdens, it markedly improved survival in co-infected chicks (Fig. 11D). Co-infection was also associated with marked leukocyte depletion, increased serum dsDNA, and elevated systemic inflammatory mediators, all of which were partially reversed by DNase I (Fig. 11E–L). Together, these findings indicate that MG infection reprograms neutrophil responsiveness and creates conditions that permit exaggerated NET-associated inflammatory pathology during secondary challenge.

## 4 Discussion

This study identifies a Ca^2+^-dependent redox–mitochondrial remodeling pathway that contributes to PAD-associated NETosis during respiratory mycoplasma infection. Rather than being considered the sole or dominant driver, mitophagy-associated remodeling is presented as a contributory module that connects mitochondrial redox disruption with PAD3 nuclear redistribution, histone citrullination, and chromatin execution in directly MG-stimulated neutrophils. At the extracellular level, MG-associated nuclease activity degrades trap DNA, reduces visible trap accumulation, and modifies the inflammatory outcomes of trap-derived material. This dual regulation suggests that NET biology during MG infection is shaped not only by the signaling events initiating NET execution, but also by pathogen-mediated remodeling of extracellular trap fate after release.

A major feature of MG-induced NET formation was an early extracellular Ca^2+^ influx ROS axis. Previous studies have already identified cytosolic Ca^2+^ mobilization as an important event regulating neutrophil oxidative metabolism [56]. In our model, MG increased intracellular Ca^2+^ and ROS levels, whereas Ca^2+^ chelation decreased ROS accumulation, extracellular DNA release, NET-like structures, and NET-associated protein abundance. These findings indicate that Ca^2+^ signaling acts upstream of ROS-associated oxidative activation and NET execution during MG challenge. Similar Ca^2+^/ROS-linked mechanisms have been described in NET formation induced by different stimuli, including PMA, LPS, parasitic stimulation, high-glucose conditions, and inflammatory cytokines, suggesting that the Ca^2+^/ROS axis represents a recurring regulatory module in NET biology rather than an MG-specific event [[57], [58], [59]]. Moreover, our data further suggest that extracellular Ca^2+^ availability has particular importance in the MG model. EGTA produced stronger inhibitory effects than intracellular Ca^2+^ chelation, and disruption of ORAI1/STIM1-associated Ca^2+^ entry reduced Ca^2+^ signals, ROS accumulation, NADPH-related activity, S100A8/9 expression, and NET release. Notably, S100A8/9 is a Ca^2+^-binding inflammatory protein and may contribute to local Ca^2+^ buffering or feedback regulation [60]. However, during sustained Ca^2+^ influx during MG stimulation, this possible buffering effect may be insufficient to counterbalance the overall Ca^2+^/ROS-driven activation state. Although these data do not establish ORAI1/STIM1 as the only pathway for Ca^2+^ entry during MG stimulation, they support a model in which MG-induced extracellular Ca^2+^ influx generates a redox-activated state that prepares neutrophils for downstream mitochondrial remodeling and PAD-associated chromatin execution.

Mitochondrial involvement in neutrophil biology has traditionally been underestimated because neutrophils contain relatively few mitochondria and depend mainly on glycolysis for energy generation [18]. Recent studies, however, have repositioned neutrophil mitochondria as important signaling organelles [19]. For example, bacterial metabolites such as *Staphylococcus aureus*-derived lactate have been reported to be sensed through neutrophil mitochondria, resulting in rapid NADPH oxidase activation and subsequent NET release [61]. In addition, the transfer of functional mitochondria through mesenchymal stromal cell-derived extracellular vesicles can improve the mitochondrial condition of neutrophils and limit NET formation [62]. Therefore, growing evidence suggests that mitochondrial status may participate in NET execution.

Consistent with the early proteomic signature, MG induced mitochondrial dysfunction and mitophagy-associated remodeling before marked NET release. Mito-TEMPO reduced mitochondrial ROS accumulation, lowered total ROS levels, partly restored mitochondrial fluorescence intensity, and relieved mitochondrial damage and mitophagy-like ultrastructural changes. These observations support a role for mitochondrial ROS in MG-induced mitochondrial remodeling, but they do not establish mitochondrial ROS as the sole trigger of mitophagy-associated alterations. Functionally, CCCP-induced mitochondrial depolarization and mitophagy-associated activation enhanced NET-associated responses, whereas Mdivi-1 weakened MG-induced extracellular DNA release and decreased CitH3, PAD3, and NE abundance. Mechanistically, mitophagy is closely related to intracellular ROS homeostasis [63], while ROS production is recognized both as a direct antimicrobial mechanism and as a central event in NET release [64]. Under MG stimulation, neutrophils generated large amounts of ROS, including mitochondrial ROS, which may cause mitochondrial membrane depolarization and thereby induce mitophagy as a compensatory response to oxidative stress. If this remodeling becomes prolonged or excessive, it may further impair mitochondrial abundance, mitochondrial function, and intracellular redox balance, thereby forming a feed-forward loop involving mitochondrial injury, ROS accumulation, and mitophagy activation [65,66]. Importantly, Mdivi-1 only partly suppressed NET release, whereas PAD inhibition abolished NETosis under both MG and CCCP conditions with limited effects on LC3 signals. Together, these findings suggest that mitophagy-associated remodeling contributes to MG-induced NET formation within a broader Ca^2+^/ROS/PAD-associated program, rather than functioning as an independent or dominant driver of MG-induced NETosis.

Beyond intracellular NET execution, our study also emphasizes the importance of extracellular trap fate after release. Previous studies have shown that *Mycoplasma hyopneumoniae* can degrade macrophage extracellular traps through nucleases and use the resulting degradation products [67]. In our experiments, EdU tracing showed that MG degrades macrophage extracellular traps in a nuclease-dependent manner and acquires DNA signals derived from these traps. Although some studies suggest that mycoplasmas benefit from exogenous nucleotide supplementation [68,69], current evidence is insufficient to directly demonstrate that MG uses trap-derived degradation products (e.g., nucleotides) as nutrient sources to promote its own replication. Therefore, the possibility that MG gains nutritional benefit from trap-derived nucleic acids remains a plausible hypothesis requiring experimental confirmation. At the same time, experiments in HD11 macrophages and tracheal epithelial cells show that intact extracellular traps are not immunologically inert. From an immunological perspective, intact extracellular traps promoted inflammatory activation, favored M1-like polarization, enhanced phagocytic and degradative functions, shifted epithelial cell death toward pyroptosis, and amplified inflammatory mediator production. Accordingly, MG-mediated NET degradation may serve not only as an immune evasion mechanism, but also as a way to regulate the local inflammatory consequences of NET accumulation. This interpretation may help explain how a chronic respiratory pathogen can reduce local NET persistence through nuclease activity and modulate the downstream inflammatory consequences of NET accumulation.

The inducible nitric oxide synthase/nitric oxide (iNOS/NO) axis may add another regulatory layer for the local NET-associated inflammatory network during MG infection. In the present study, transcriptomic profiling of MG-infected CETs showed activation of innate immune and inflammatory pathways, including NF-κB, Toll-like receptor, MAPK, NOD-like receptor, and inflammatory response pathways, indicating that epithelial cells have an important role in shaping the local inflammatory microenvironment. Consistent with previous findings [44], MG infection promoted M1 polarization and enhanced iNOS-associated responses in HD11 macrophages, suggesting that macrophage-derived NO may contribute to local inflammation. NO has both pro- and anti-inflammatory activities, can diffuse across cell membranes, and is increased in various infection models, thereby contributing to disease progression [70,71]. Notably, previous studies have reported that iNOS can promote NET formation [72], whereas inhibition of nitric oxide synthase (NOS inhibitors) can reduce NET release [73]. Therefore, the iNOS/NO axis may affect NET-associated responses by modifying the local inflammatory microenvironment, particularly under co-infection or excessive inflammatory activation conditions. This possibility provides a tissue-level regulatory dimension for NET biology during MG infection and may contribute to the enhanced NET release observed during co-infection.

The *in vivo* experiments further evaluated local NET-related responses and systemic neutrophil priming during MG infection. NET-associated signals were detected in the trachea during infection, supporting the relevance of neutrophil activation at the primary colonization site [74]. More importantly, neutrophils isolated from MG-infected chicks showed enhanced NET release after secondary LPS stimulation, indicating that previous MG infection is associated with a primed or reprogrammed neutrophil state. Notably, this *in vivo* phenotype was more closely linked to increased ROS accumulation and MAPK activation than to the mitophagy-associated alterations observed *in vitro*. This *in vivo* phenotype is also consistent with a two-hit model of NET formation [75,76], in which MG infection sensitizes circulating neutrophils to respond excessively to a secondary challenge. This difference is informative rather than contradictory, because it suggests that the *in vivo* response reflects broader inflammatory reprogramming by the host milieu, including inflammatory mediators known to regulate neutrophil activation and NET release [77,78], rather than a simple reproduction of the direct intracellular mechanisms observed in isolated neutrophils. The MG-*E. coli* co-infection model further supports the pathological importance of this priming, as co-infection increased serum dsDNA and inflammatory mediators, whereas DNase I partly improved survival and reduced the inflammatory burden. Although DNase I cannot separate NET-derived DNA from other extracellular DNA sources, these results suggest that excessive extracellular DNA/NET-associated burden contributes to disease severity during secondary infection. Together, these findings indicate that MG infection promotes local NET-related responses and ROS/MAPK-linked neutrophil priming, thereby increasing the risk of excessive extracellular DNA/NET-associated inflammation during secondary challenge.

Despite the mechanistic model presented here, several limitations should be recognized. First, multiple key nodes identified in this study were characterized mainly through pharmacological perturbation. Although EGTA/BAPTA, 2-APB, CCCP, Mdivi-1, PAD inhibition, and ATA are commonly used, their specificity and selectivity are not complete. Therefore, these results support the involvement of these pathways in MG-induced NETosis but do not provide definitive genetic evidence of causality. ATA-preserved NET structures should also be interpreted with caution, because ATA may influence neutrophil activation or MG behavior beyond nuclease inhibition. Second, the difference between the *in vitro* and *in vivo* findings suggests that MG may regulate NET biology through context-dependent modules. The mechanisms of MG-induced NET release *in vivo*, the link between mitophagy and PAD-associated execution, and the metabolic fate of DNA products generated by nuclease remain partly unclear. Future studies using more advanced gene-editing tools in animal models and mycoplasma systems will be needed to clarify these issues. Despite these limitations, the present indicate that MG reshapes NET biology at several levels, including induction, execution, degradation, and downstream inflammatory effects.

## 5 In conclusion

In summary, this study identified a dual regulatory mechanism by which MG affects neutrophil NET biology. MG induces a Ca^2+^/ROS-related intracellular remodeling program involving mitochondrial dysfunction, mitochondrial ROS accumulation, mitophagy-related remodeling, and PAD3-associated chromatin execution, thereby promoting NET release. Meanwhile, MG-associated nuclease activity degrades extracellular trap DNA and changes the persistence and inflammatory activity of trap-derived material. These findings suggest that Ca^2+^ signaling, mitochondrial remodeling, PAD-associated chromatin execution, pathogen nuclease activity, and extracellular DNA/NET burden may serve as potential targets for future therapeutic strategies against respiratory mycoplasma-associated inflammation and co-infection pathology.

## CRediT authorship contribution statement

**Shun Wang:** Conceptualization, Methodology, Project administration, Writing – original draft. **Weiqi Liu:** Data curation, Methodology, Writing – original draft. **Fuhua Gu:** Data curation, Formal analysis, Investigation. **Jian Wang:** Conceptualization, Methodology, Validation. **Yuquan Guo:** Formal analysis, Methodology. **Liyang Guo:** Software, Visualization. **Yifan Li:** Investigation, Validation. **Kexin Wang:** Investigation, Methodology. **Jie Zhang:** Data curation, Formal analysis. **Yecheng Yao:** Supervision. **Zhiyong Wu:** Funding acquisition, Project administration, Writing – review & editing. **Jichang Li:** Funding acquisition, Investigation, Project administration, Supervision, Writing – review & editing.

## Declaration of competing interest

The authors declare that the research was conducted in the absence of any commercial or financial relationships that could be construed as a potential conflict of interest.

## Data Availability

Data will be made available on request.
