# Supplementary material for: Calcium-dependent redox signaling connects mitochondrial remodeling with PAD-associated NETosis during respiratory mycoplasma infection
Source: Redox Biol. 2026 Jul 18;96:104316. doi: 10.1016/j.redox.2026.104316 (PMC13393679; doi:10.1016/j.redox.2026.104316)
Supplement: Multimedia component 1 [file mmc1.docx]

**Calcium-dependent redox signaling connects mitochondrial remodeling with PAD-associated NETosis during respiratory mycoplasma infection**

**Shun Wang^#^, Weiqi Liu^#^, Fuhua Gu, Jian Wang, Yuquan Guo,**

**Liyang Guo, Yifan Li, Kexin Wang, Jie Zhang, Yecheng Yao**

**Zhiyong Wu*, Jichang Li***

College of Veterinary Medicine, Northeast Agricultural University, 600 Changjiang Road, Xiangfang District, Harbin 150030, P. R. China; Heilongjiang Key Laboratory for Animal Disease Control and Pharmaceutical Development, 600 Changjiang Road, Xiangfang District, Harbin 150030, P. R. China

^#^ These authors contributed equally to this work

^*^Corresponding authors at College of Veterinary Medicine, Northeast Agricultural University, 600 Changjiang Road, Xiangfang District, Harbin 150030, P. R. China

E-mail: wuzhiyong@neau.edu.cn (Z. Wu); lijichang@neau.edu.cn (J. Li)

**Supplementary Materials**

**Materials and methods**

**Grouping and Processing for HD11 cells Experiments**

To examine the intracellular localization of NETs in HD11 cells, cells were divided into two groups: CG and NETs (50 ng/mL). Cells were seeded in confocal dishes and allowed to reach 50%–70% confluence before treatment with an equal volume of PBS or NETs for 4 h, followed by sample collection.

To induce macrophage extracellular traps (METs), HD11 cells were divided into two groups: CG and PMA group. When cell confluence reached 30%–50%, cells were stimulated with PBS or PMA (300 nM) for 3 h and then collected for subsequent analyses.

To determine whether MG could degrade METs, HD11 cells were divided into four groups: CG, PMA, PMA + MG, and PMA + MG + ATA. Briefly, cells were first incubated with EdU (C0078S, Beyotime) for 4 h to label newly synthesized DNA. Cells were then treated with PBS or PMA (300 nM) for 3 h to induce EdU-labeled MET formation. Subsequently, cells were exposed to PBS, MG (100 MOI), or MG plus ATA (25 μM) for an additional 2 h before further analyses.

To assess whether MG uptake of extracellular nucleotide-like substrates was associated with nuclease activity, two groups were included: MG and MG + EDTA. Briefly, newly synthesized DNA in HD11 cells was first labeled with EdU, followed by PMA stimulation to generate EdU-labeled METs. Cells were then gently washed, and MET-containing supernatants were collected by gentle pipetting in MG medium. The supernatants were centrifuged at 1000 × g for 10 min to remove cellular debris. The resulting supernatants were then incubated in the presence or absence of EDTA (5 mM), which was used to chelate metal ions and thereby inhibit nuclease activity, before co-incubation with PBS-washed third-passage MG for 12 h.

**Grouping and Processing for Chicken Neutrophil Experiments**

To determine the appropriate MG challenge dose, neutrophils were assigned to six groups: control group (CG), positive control group (PMA, 2.5 μM), and MG groups challenged at MOIs of 20, 40, 80, or 160. Cells were stimulated for 3 h and then collected for subsequent analyses.

To assess the effects of MG on chicken neutrophils and NET formation, neutrophils were divided into two groups: CG and MG group (80 MOI). Where indicated for visualization of NET structures, cells were pretreated with ATA (25 μM) before MG stimulation to inhibit MG-associated nuclease activity. Cells were then stimulated for 3 h and subjected to the indicated assays.

To analyze early proteomic changes induced by MG, neutrophils were divided into two groups: CG and MG group (80 MOI). Cells were treated with PBS or MG for 1 h, collected, and submitted to Sangon Biotech (Shanghai, China) for proteomic analysis.

To evaluate the role of Ca²⁺ in MG-induced NET formation, neutrophils were treated under different grouping schemes depending on the downstream assay. To compare the relative contributions of extracellular and intracellular Ca²⁺, cells were divided into three groups: MG, MG + EGTA, and MG + BAPTA. EGTA (10 μM) and BAPTA (25 μM) were applied 1 min and 30 min before MG stimulation, respectively, to chelate extracellular or intracellular Ca²⁺, followed by MG challenge for 3 h. In some assays, CG and inhibitor-alone groups were also included to assess the effects of the chelators alone.

To explore the potential mechanism underlying MG-induced Ca²⁺ influx, neutrophils were divided into two groups, CG and MG group (80 MOI), and used for detection of ORAI1-, STIM1-, and S100A8/9-associated signals.

To evaluate the role of ORAI1/STIM1-associated Ca²⁺ entry in MG-induced NET formation, neutrophils were divided into four groups: CG, 2-APB group (50 μM), MG group (80 MOI), and MG + 2-APB group. Cells were pretreated with 2-APB for 1 h to block Ca²⁺ entry and then challenged with MG for 3 h. For inhibitor or activator treatments, the corresponding vehicle controls were included where applicable, and the final solvent concentration was kept consistent among groups.

To examine the effect of mitophagy activation on NET formation, neutrophils were divided into three groups: CG, CCCP group (10 μM), and MG group (80 MOI). CG cells were treated with PBS, CCCP-treated cells were exposed for 2 h, and MG-treated cells were challenged for 3 h before subsequent analyses.

To investigate the potential role of mitophagy in MG-induced NET formation, neutrophils were divided into four groups: CG, Mdivi-1 group (20 μM), MG group (80 MOI), and MG + Mdivi-1 group. Cells were pretreated with Mdivi-1 for 2 h and then challenged with MG for 3 h.

To explore whether mitophagy-associated regulation affects NET release through PAD-dependent mechanisms, cells were assigned to the following groups: CG, MG+Mdivi-1 (20 μM), MG (80 MOI), and CCCP (10 μM). The subcellular distribution of PAD3 was assessed at 1, 2, and 3 h after treatment. Since CCCP was applied for a total of 2 h, untreated cells were defined as the 1 h reference point, and cells exposed to CCCP for 1 h were considered the 2 h time point.

To determine whether mitophagy could independently induce NET formation, neutrophils were divided into five groups: CG, MG, MG + Cl-amidine, CCCP, and CCCP + Cl-amidine. The PAD inhibitor Cl-amidine (5 μM) was added 30 min before stimulation, followed by treatment with CCCP for 2 h or MG for 3 h.

**Cytokine measurements**

The levels of cytokines in tissue samples, cell culture supernatants, and blood-derived samples were measured using commercial ELISA kits according to the manufacturers’ instructions. The analyzed cytokines included IL-1β (AD0457Ch), IL-6 (AD0451Ch), and TNF-α (AD0003Ch) from Chenglin Biotechnology Co., Ltd.; CXCL1 (MM-60082O2) and CXCL2 (MM-60083O2) from Jiangsu Meimian Industrial Co., Ltd.; and CRP (JLC-Y4141) from Jinghang Biotechnology Co., Ltd. Briefly, cell culture supernatants, tissue homogenate supernatants, or serum samples were added to ELISA plates together with the corresponding standards. After incubation, washing, and color development according to the kit protocols, absorbance was measured using a microplate reader, and cytokine concentrations were calculated based on standard curves.

NADPH-related activity was measured using a commercial assay kit according to the manufacturer’s instructions (S0179, Beyotime). Briefly, after the indicated treatments, cells were collected and lysed as required by the kit protocol, and the resulting supernatants were used for analysis. Samples were mixed with the corresponding reaction working solution and incubated at 37°C, and absorbance was measured at 450 nm using a microplate reader. NADPH-related activity was calculated based on a standard curve and used as a complementary indicator of oxidant-associated signaling.

**Nuclear and cytoplasmic protein extraction**

To assess the relative distribution of PAD3 protein in the nucleus and cytoplasm, nuclear and cytoplasmic proteins were extracted from differently treated cells. Briefly, fractionation was performed using a Nuclear and Cytoplasmic Protein Extraction Kit (P0028, Beyotime) according to the manufacturer’s instructions. Cells were washed with ice-cold PBS, gently harvested with a cell scraper, and collected by centrifugation. The resulting pellet was incubated with cytoplasmic protein extraction buffer, and the supernatant was collected after centrifugation as the cytoplasmic protein fraction. The remaining pellet was then lysed with nuclear extraction buffer to obtain the nuclear protein fraction.

**Reactive oxygen species (ROS) detection**

After the indicated treatments, intracellular ROS levels were measured using a reactive oxygen species assay kit (S0033S, Beyotime) according to the manufacturer’s protocol. Cells were incubated with the working solution at 37°C in the dark, gently washed to remove excess probe, and then subjected to fluorescence detection. ROS signals were visualized using fluorescence microscopy or flow cytometry, and fluorescence intensity was quantified using a fluorescence microplate reader where indicated. To reduce overinterpretation of probe-based ROS measurements, these readouts were interpreted in the context of Ca²⁺ modulation, NADPH-related activity, mitochondrial membrane potential, mitochondrial abundance, mitochondrial ROS accumulation, mitophagy-associated markers and NET-associated outputs.

**Fluo-4 calcium fluorescence assay**

After the indicated treatments, intracellular Ca²⁺ levels were measured using a Fluo-4 calcium assay kit (E-BC-F100, Elabscience Biotechnology Co., Ltd.) according to the manufacturer’s instructions. Briefly, cells were incubated with the Fluo-4 working solution at 37°C in the dark, gently washed, and allowed to equilibrate before analysis. Fluorescence signals were then detected using a fluorescence microscope or quantified by flow cytometry under the instrument settings recommended by the manufacturer.

**Western blotting (WB)**

After the indicated treatments, cells were washed with ice-cold PBS and lysed in RIPA buffer containing PMSF. Cells attached to the coverslips were gently scraped and collected, and lysates were centrifuged at 4°C to remove insoluble debris. Protein concentrations were determined using a BCA protein assay kit. Equal amounts of protein were mixed with 5× SDS loading buffer, boiled for 10 min, separated by SDS-PAGE, and transferred onto PVDF membranes. Membranes were blocked in blocking buffer, incubated with the indicated primary antibodies overnight at 4°C, washed with TBST, and then incubated with HRP-conjugated secondary antibodies at room temperature. Protein bands were visualized using enhanced chemiluminescence reagents. Antibody sources and working dilutions are listed in the Supplementary Materials.

**Antibody sources and working dilutions**

Antibodies were purchased from the following companies: Abmart (Shanghai, China), Abcam (Shanghai, China), Sangon Biotech (Shanghai, China), Wanleibio (Shenyang, China), and Bioss (Beijing, China). Catalog numbers and working dilutions for Western blotting (WB) and immunofluorescence (IF) are listed below.

CitH3 (Abcam, ab281584) (1:1000, WB; 1:500, IF); NE (Abcam, ab131260) (1:1000, WB; 1:100, IF); PAD3 (Abcam, ab50246) (1:1000, WB; 1:100, IF); β-actin (Abmart, P30002S) (1:2000, WB); HSP60 (Bioss, bs-0191R) (1:1000, WB); TOMM20 (Bioss, bs-7357R) (1:1000, WB; 1:100, IF); LC3 (Abmart, T55992S) (1:1000, WB; 1:100, IF); P62, (Abmart, T55546F) (1:1000, WB); Parkin (Wanleibio, WL02512) (1:1000, WB); PINK1 (Wanleibio, WL04963) (1:1000, WB); Lamin B1 (Wanleibio, WL01775) (1:1000, WB); ORAI1 (Bioss, bsm-61611R) (1:1000, WB); STIM1 (Bioss, bsm-61325R) (1:1000, WB); S100A8/9 (Bioss, bs-41227R) (1:1000, WB); P38 (Wanleibio, WL00764) (1:1000, WB); p-P38 (Wanleibio, WLP1576) (1:1000, WB); JNK (Wanleibio, WL01295) (1:1000, WB); p-JNK (Wanleibio, WL01813) (1:1000, WB); ERK (Bioss, bsm-52259R) (1:1000, WB); p-ERK (Bioss, bsm-54491R) (1:1000, WB); CD86 (Sangon Biotech, D290249) (1:1000, WB); iNOS (Sangon Biotech, D362846) (1:1000, WB); IL-1β (Wanleibio, WL02257) (1:1000, WB); TNF-α (Wanleibio, WL01581) (1:1000, WB); cGAS (Abmart, PQA3430) (1:1000, WB; 1:100, IF); STING (Abmart, MA8145) (1:1000, WB; 1:100, IF).

**Ultrastructural observation, histopathology, and immunofluorescence staining**

Collected cells and tracheal tissue samples were fixed in either 2.5% glutaraldehyde or 4% paraformaldehyde, as appropriate. Samples used for scanning electron microscopy (SEM) and transmission electron microscopy (TEM) were further post-fixed in 1% osmium tetroxide and dehydrated through graded ethanol and acetone. For SEM analysis, dehydrated samples were dried, coated with a conductive metal layer using an ion sputter coater, and examined with a scanning electron microscope (SU8010, HITACHI, Japan). For TEM analysis, samples were embedded in epoxy resin, sectioned into ultrathin sections, stained with uranyl acetate and lead citrate, and then examined using a transmission electron microscope (JEOL Ltd., Tokyo, Japan). For histopathological examination, samples were dehydrated through graded ethanol, embedded in paraffin, sectioned, and stained with hematoxylin and eosin (H&E). Histological changes were then evaluated under a light microscope.

For immunofluorescence staining of fixed cells and tissues, samples were permeabilized with 0.1% Triton X-100 and blocked with 3% bovine serum albumin (BSA) (CA1381, Coolaber Science & Technology). Samples were then incubated with the indicated primary antibodies overnight at 4°C. After washing, samples were incubated with the fluorescent secondary antibody (AB205718, Abcam) for 1 h at room temperature. Where indicated, nuclei or cytoskeletal structures were counterstained with DAPI (PR01140, Medlife, Shanghai, China), Actin-Tracker Green (C2232, Beyotime), or Sytox dye. Images were acquired using a fluorescence microscope or a laser confocal microscope. Detailed information on primary antibodies and dilution ratios is provided in the Supplementary Materials.

**Assessment of mitochondrial abundance and function**

To assess mitochondrial abundance, neutrophils were stained with MitoTracker Deep Red probe (C1032, Beyotime) after the indicated treatments and incubated according to the manufacturer’s instructions. Fluorescence signals were then observed using a laser confocal microscope. To evaluate mitochondrial membrane potential, cells were stained using a mitochondrial membrane potential assay kit (E-CK-A301, Elabscience Biotechnology Co., Ltd.) and analyzed by flow cytometry according to the manufacturer’s instructions. To assess mitochondrial ROS-associated signals, neutrophils were stained with the mitochondrial superoxide indicator MitoSOX™ Red (S0061S, Beyotime) according to the manufacturer’s protocol. Fluorescence images were acquired using an inverted fluorescence microscope under identical exposure settings across groups. MitoSOX™ Red fluorescence intensity was quantified using ImageJ software and used as an indicator of mitochondrial superoxide accumulation in the indicated groups.

**Mass spectrometric analysis of MG-induced NET-enriched fractions**

To characterize the protein composition of MG-induced NETs, NET-enriched fractions were collected from neutrophils after MG stimulation and subjected to mass spectrometry analysis. Briefly, after stimulation under the indicated conditions, culture supernatants were gently removed, and NET-associated material attached to the coverslips was recovered by gentle pipetting with ice-cold PBS. The collected suspensions were sequentially centrifuged at low speed to remove intact cells and large cellular debris, followed by an additional centrifugation step to reduce potential contamination by residual MG organisms. The precleared supernatants were then centrifuged at 4°C and 18000 × g for 15 min to obtain NET-enriched pellets. The resulting NET-enriched fractions were submitted to Sangon Biotech (Shanghai, China) for mass spectrometric analysis.

**Acridine Orange (AO)/Ethidium Bromide (EB) staining**

To distinguish viable and dead cells after the indicated treatments, neutrophils were stained using an AO/EB staining kit (DA0039, Leagene) according to the manufacturer’s instructions and observed under a fluorescence microscope.

**Cell viability assay**

To assess the effects of MG or NETs on cell viability, a Cell Counting Kit-8 (CCK-8; C0038, Beyotime) assay was performed. After the indicated treatments, cells were incubated with CCK-8 working solution, and absorbance was measured using a microplate reader to evaluate cell viability.

**TdT-mediated dUTP nick end labeling assay**

To assess apoptotic cell death in CETs after MG and NET treatment, a TdT-mediated dUTP nick end labeling (TUNEL) assay was performed according to the manufacturer’s instructions (E-CK-A320, Elabscience, China). Briefly, after the indicated treatments, cells were fixed and permeabilized with 0.1% Triton X-100, followed by incubation with the TUNEL reaction mixture in the dark. After washing, nuclei were counterstained with DAPI, and images were acquired using an inverted fluorescence microscope. TUNEL-positive signals were interpreted as apoptotic cells with DNA fragmentation.

**DNA hydrolysis assay**

To determine whether MG possesses nuclease activity and whether ATA inhibits MG-associated nuclease activity, MG suspensions or culture supernatants were co-incubated with λDNA and the DNA degradation pattern was evaluated. Briefly, MG suspensions at different concentrations (1 × 10^^6^–1 × 10^^9^ CCU/mL), equal volumes of MG culture supernatant, MG (1 × 10^^6^ CCU/mL) plus ATA (25 μM), and a DNase I positive control group (2 U; D7076, Beyotime) were incubated with λDNA (200 ng; B610010, Sangon Biotech) in Tris-HCl buffer (pH 7.5) at 37°C for 30 min. After incubation, samples were resolved on 1% agarose gels, and DNA hydrolysis was assessed based on the resulting band patterns.

**Results**

**
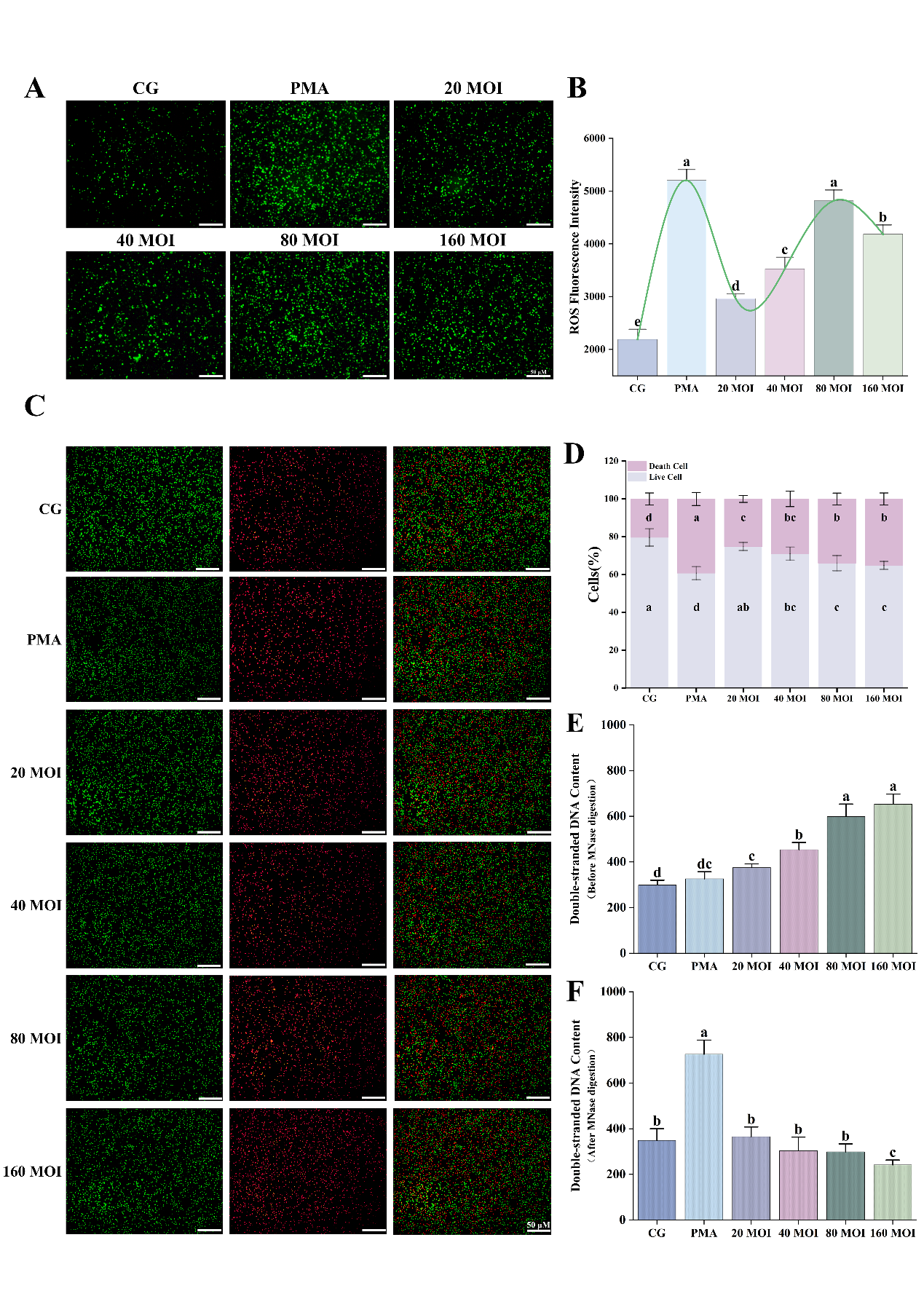
**

**Fig. S1. Dose selection of MG infection and its effects on oxidative stress and cell status in neutrophils.** (A) Representative fluorescence images showing intracellular ROS signals in neutrophils stimulated with different concentrations of MG (20, 40, 80, and 160 MOI) or PMA (2.5 μM) for 3 h (n=3). (B) Quantification of ROS fluorescence intensity in neutrophils after stimulation with the indicated concentrations of MG or PMA (n=6). (C–D) Representative AO/EB staining images and corresponding quantification of neutrophils after stimulation with different concentrations of MG or PMA (n=6). (E–F) Schematic presentation and quantification of dsDNA levels in neutrophils after stimulation with MG or PMA, with or without micrococcal nuclease digestion (n=6). Data are presented as means ± SD from at least three independent experiments. Different lowercase letters indicate statistically significant differences among groups (P < 0.05).

**Fig. S2. Visualization of NETs.** (A) Representative scanning electron microscopy (SEM) images of neutrophils after stimulation with different concentrations of MG (20, 40, 80, and 160 MOI) or PMA (2.5 μM) for 3 h (n=3). (B) Sytox Green staining showing NET release from neutrophils. Red arrows indicate NETs (n=3).


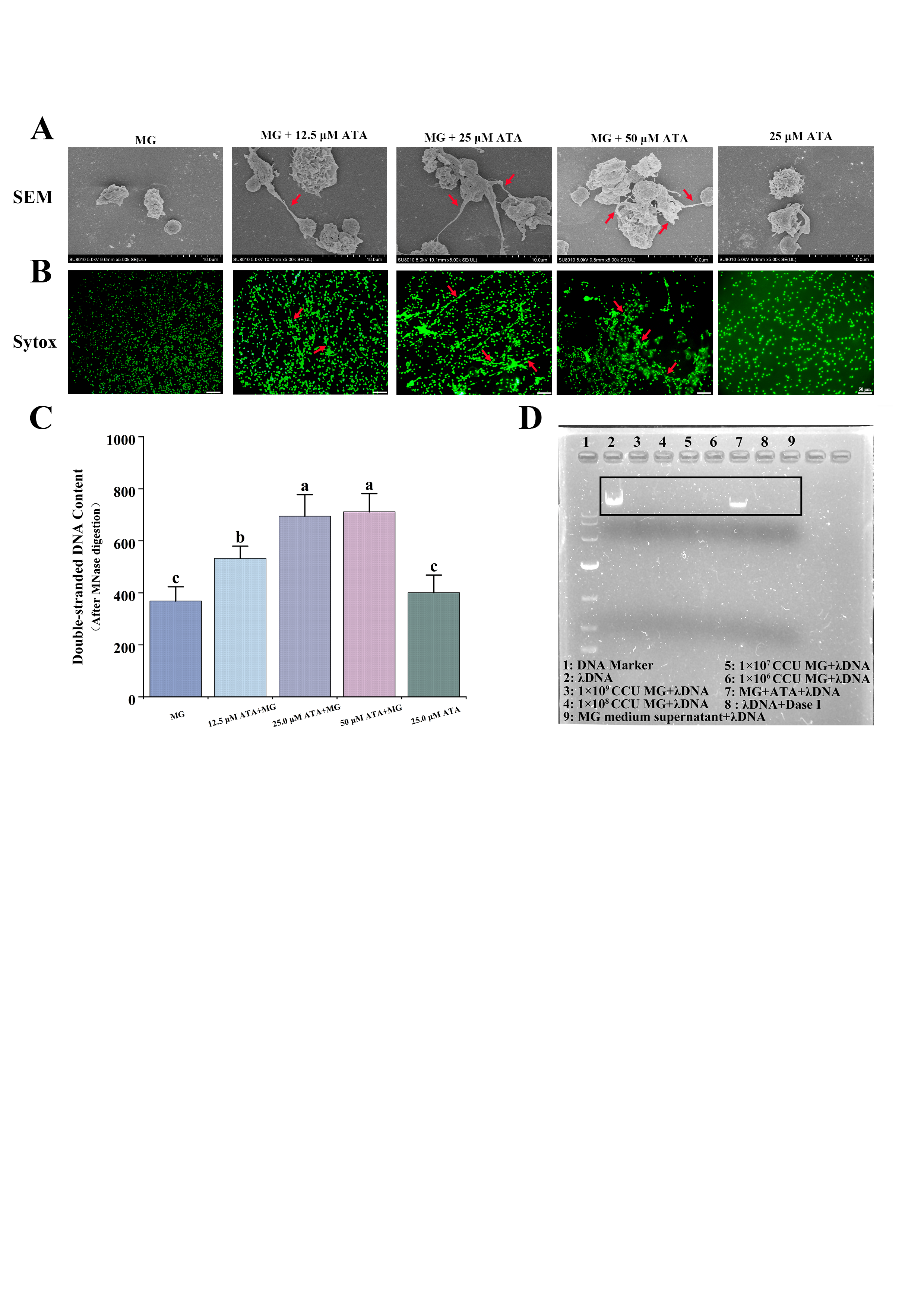


**Fig. S3. ATA dose optimization and its effects on MG-associated nuclease activity and NET visualization.** (A) Representative scanning electron microscopy (SEM) images of neutrophils pretreated with different concentrations of ATA (0, 12.5, 25, and 50 μM) before MG stimulation for 3 h. Red arrows indicate NETs (n=3). (B) Sytox Green staining showing extracellular DNA signals in neutrophils after pretreatment with different concentrations of ATA followed by MG stimulation. Red arrows indicate NETs (n=3). (C) Quantification of extracellular dsDNA levels induced by MG after treatment with different concentrations of ATA (n=6). Samples were digested with micrococcal nuclease (2 gel units) at 37°C for 30 min before measurement. (D) DNA hydrolysis assay. Different concentrations of MG, MG culture supernatant, or ATA (25 μM) were incubated with λDNA in Tris-HCl buffer (pH 7.5) at 37°C for 30 min, and DNA digestion patterns were analyzed by 1% agarose gel electrophoresis (n=3). Data are presented as means ± SD from at least three independent experiments. Different lowercase letters indicate statistically significant differences among groups (P < 0.05).


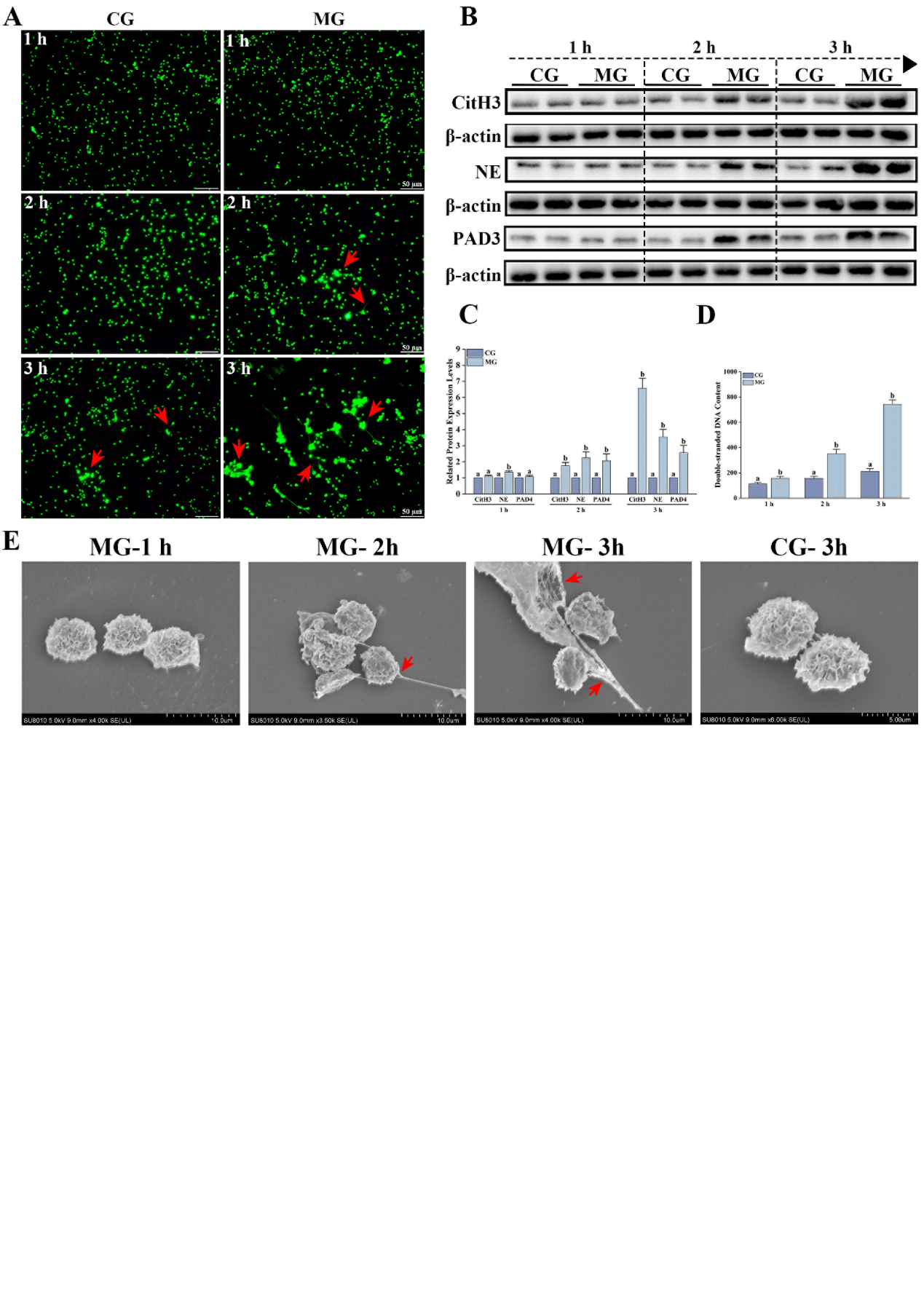


**Fig. S4. Time-course analysis of MG (80 MOI)-induced NET formation.** (A) Representative Sytox Green staining images of neutrophils at different time points after stimulation with MG (80 MOI) (n=3). Red arrows indicate NETs. (B–C) Western blot analysis and quantification of NET-associated proteins in neutrophils at different time points after MG stimulation (n=6). (D) Extracellular dsDNA levels released from neutrophils at different time points after MG stimulation (n=6). (E) Representative scanning electron microscopy (SEM) images showing NET formation at different time points after MG stimulation (n=3). Red arrows indicate NETs. Data are presented as means ± SD from at least three independent experiments. Different lowercase letters indicate statistically significant differences among groups (P < 0.05).


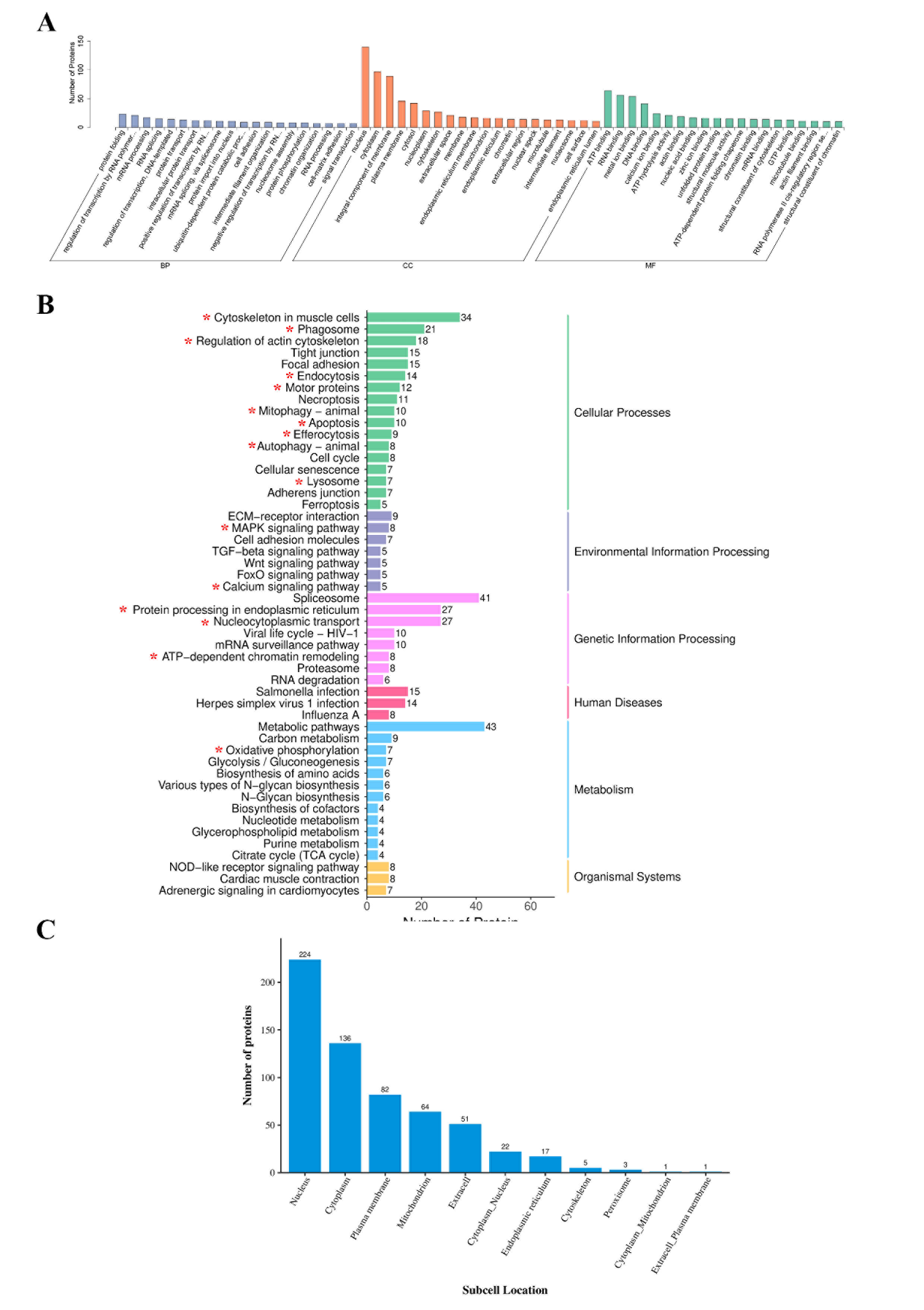


**Fig. S5. Mass spectrometric analysis of MG-induced NETs from neutrophils.** (A) Gene Ontology (GO) annotation analysis of NET-associated proteins. (B) Kyoto Encyclopedia of Genes and Genomes (KEGG) pathway annotation analysis of NET-associated proteins. Red asterisks indicate pathways that overlapped with those identified in the early proteomic analysis of neutrophils stimulated with MG (80 MOI, 3 h). (C) Subcellular localization analysis of NET-associated proteins.


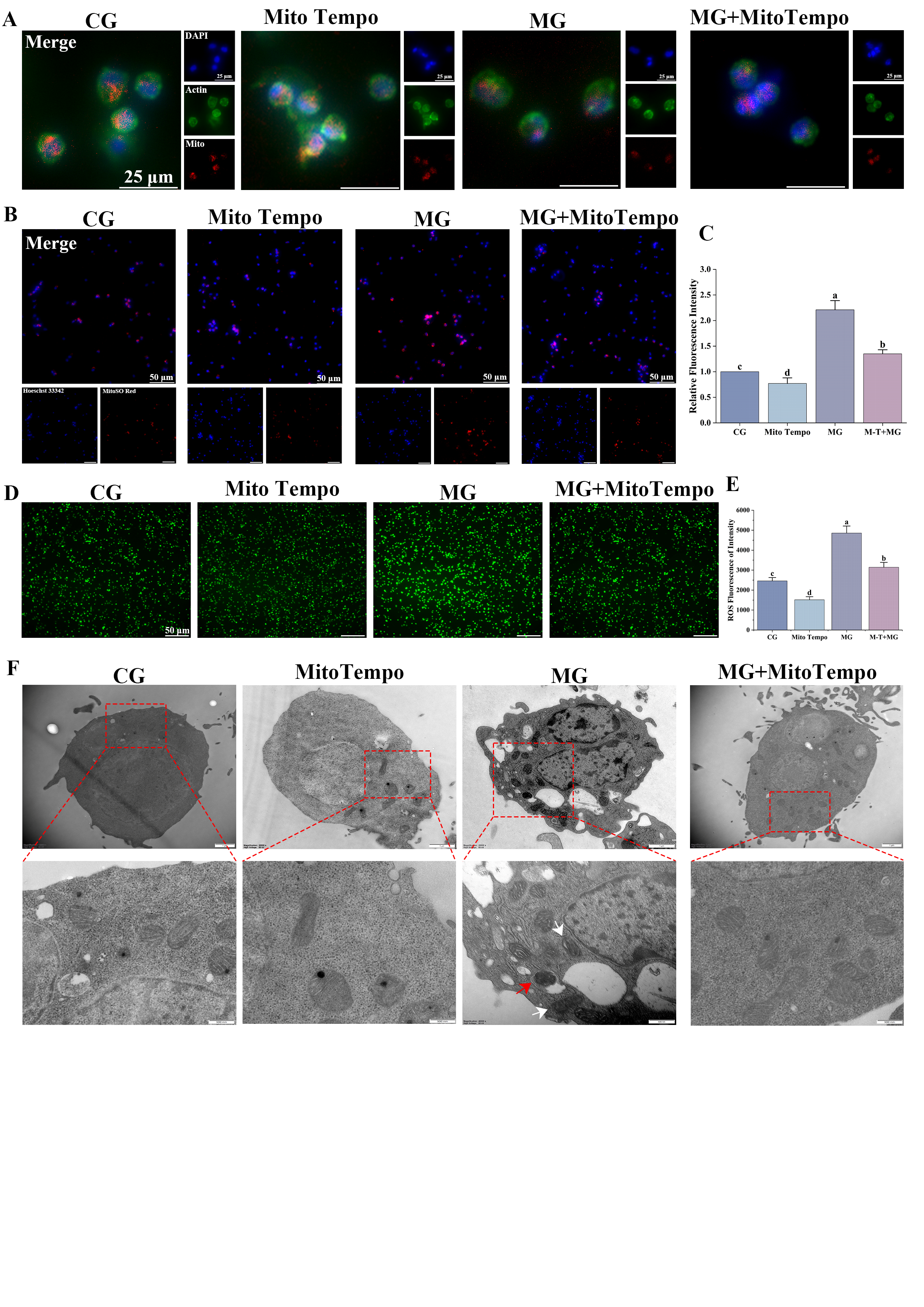


**Fig. S6. Mitochondrial ROS contributes to MG-induced mitochondrial damage and mitophagy-associated remodeling in neutrophils.** To investigate the potential role of mitochondrial ROS in MG-induced mitochondrial remodeling, neutrophils were pretreated with the mitochondria-targeted superoxide scavenger Mito-TEMPO (100 μM) for 1 h before MG challenge. (A) Representative confocal microscopy images and quantitative analysis of mitochondrial fluorescence intensity in the indicated groups (n=3). (B and C) Representative immunofluorescence images and quantitative analysis of mitochondrial ROS signals labeled with MitoSOX™ Red in the indicated groups (n=3). (D and E) Representative fluorescence microscopy images and fluorescence microplate reader-based quantification of intracellular ROS levels in the indicated groups (n=6). (F) Representative TEM images showing ultrastructural changes in neutrophils from the indicated groups (n=3). Red arrows indicate mitophagy-related structures, and white arrows indicate damaged mitochondria. Arrows indicate mitochondrial damage or autophagy-associated ultrastructural features where applicable. Data are presented as the mean ± SD from at least three independent experiments. Different lowercase letters indicate statistically significant differences among groups (P < 0.05).


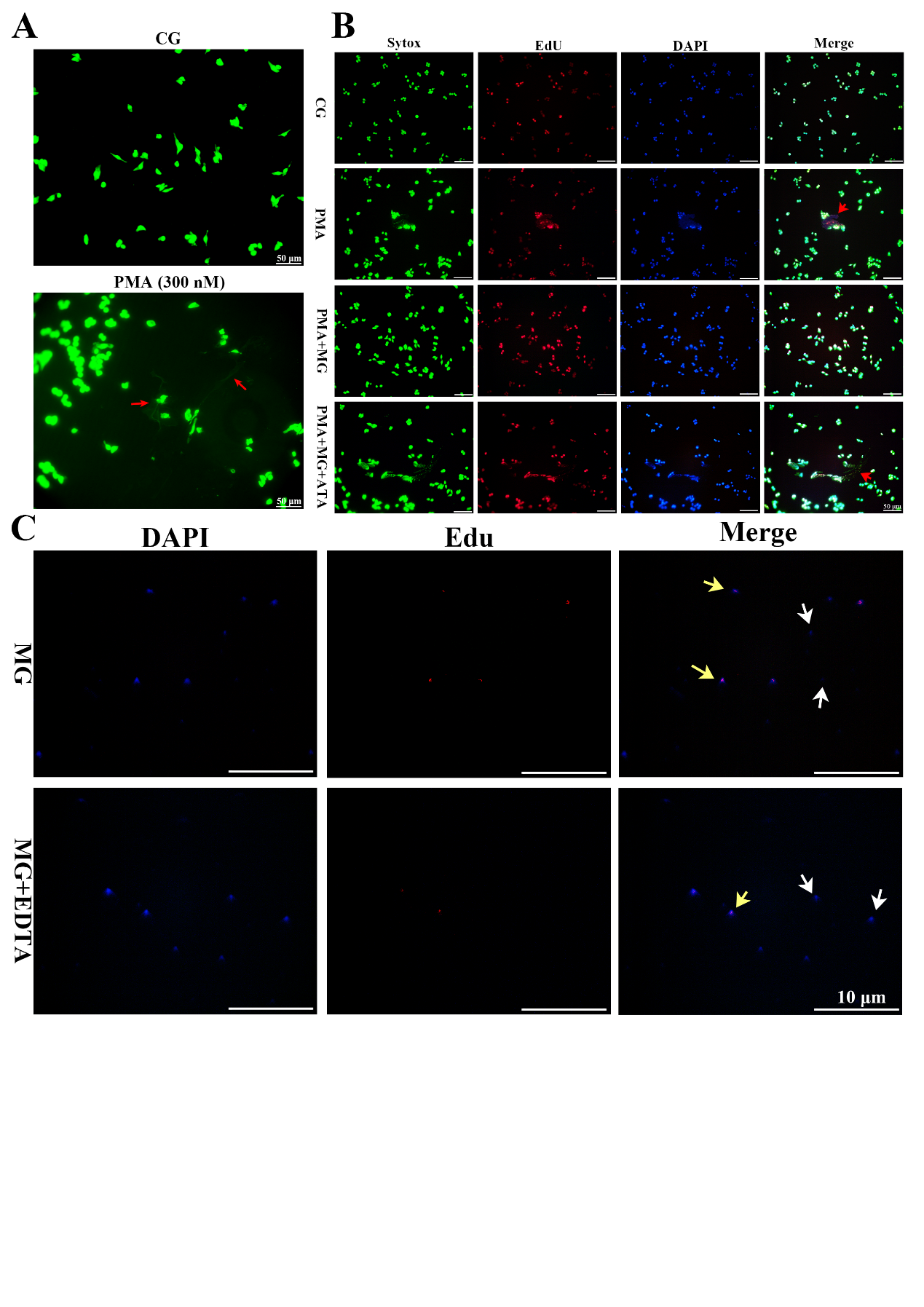


**Fig. S7. MG-associated nuclease activity degrades METs and facilitates access to MET-derived nucleic acid products.** METs were induced in HD11 macrophages by PMA stimulation (300 nM, 3 h). To assess the effect of MG-associated nuclease activity on MET integrity, HD11 cells were incubated with EdU for 4 h to label newly synthesized DNA and then stimulated with PMA (300 nM, 3 h) to generate EdU-labeled METs. MG (100 MOI) was subsequently incubated with the labeled METs for 2 h in the presence or absence of the nuclease inhibitor ATA. To evaluate whether nuclease activity contributes to acquisition of MET-derived nucleic acid products, EdU-labeled METs were isolated and incubated with MG for 12 h in EDTA-treated or untreated medium, followed by fluorescence imaging of MG-associated EdU signals. (A) Representative fluorescence microscopy images of Sytox Green-stained METs induced in HD11 macrophages (n=3). (B) Representative fluorescence microscopy images of Sytox Green-, EdU-, and DAPI-labeled METs showing the effect of MG on MET integrity (n=3). (C) Representative oil-immersion fluorescence microscopy images showing EdU-positive signals associated with MG (n=3). Yellow arrows indicate EdU-positive signals detected in MG, and white arrows indicate MG.


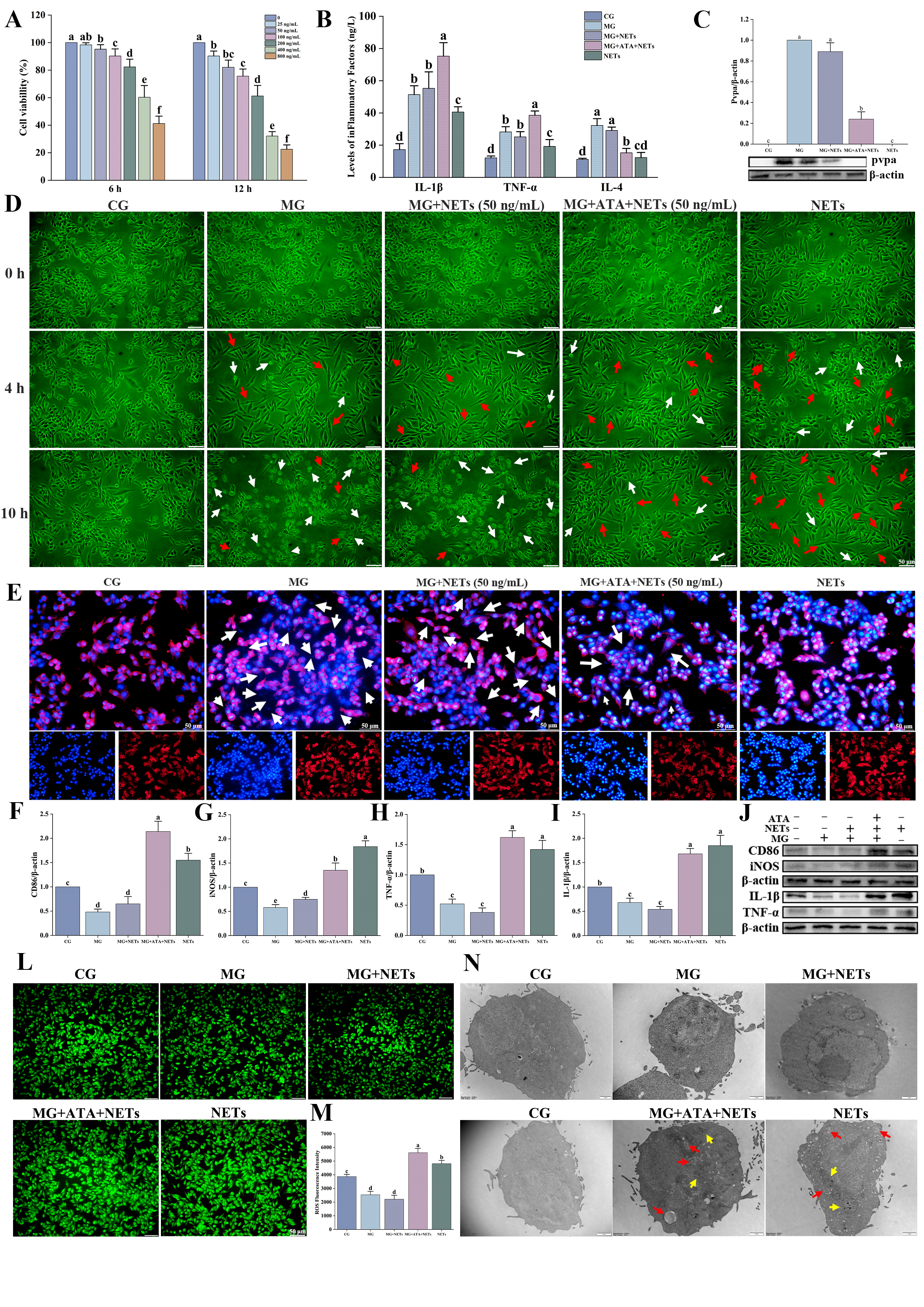


**Fig. S8. NETs protected from MG-mediated degradation promote an M1-like polarization state in HD11 cells.** To examine the effects of NETs on HD11 cells, experiments were performed based on our previously established model in which MG (400 MOI) induced a typical M1-like polarization phenotype at 4 h and a typical M2-like polarization phenotype at 10 h. CCK-8 analysis identified 50 ng/mL as the working concentration of NETs (A). HD11 cells were then assigned to five groups: CG, MG, MG + NETs, MG + ATA + NETs, and NETs. Compared with the MG and MG + NETs groups, the MG + ATA + NETs group showed increased levels of the M1-associated factors IL-1β and TNF-α and decreased levels of the M2-associated factor IL-4 (B). In parallel, MG-associated burden was reduced in the MG + ATA + NETs group (C, E). Morphological observation showed that HD11 cells co-incubated with MG displayed a typical M1-like phenotype (spindle-shaped and elongated) at 4 h but a typical M2-like phenotype (round or oval) at 10 h. In contrast, NETs protected from MG-mediated degradation promoted an M1-like morphology at both 4 h and 10 h (D). Consistently, NET treatment increased the expression of M1-associated proteins, including CD86, iNOS, IL-1β, and TNF-α (F–J). With respect to ROS, MG and MG + NETs decreased intracellular ROS levels relative to the CG group, whereas the MG + ATA + NETs and NETs groups increased ROS levels (L, M). In addition, transmission electron microscopy showed that NET treatment was associated with autophagy-related ultrastructural changes in HD11 cells (N).

(A) CCK-8 assay showing viability of HD11 cells after co-incubation with different concentrations of NETs for 6 h or 12 h (n=6). (B) ELISA analysis of IL-1β, TNF-α, and IL-4 levels in the indicated groups after 10 h of treatment (n=6). (C) Western blot analysis of the MG adhesin protein pvpA as an indicator of MG-associated burden (n=3). (D) Representative morphological images of HD11 cells in the indicated groups at 4 h and 10 h (n=3). Red arrows indicate M1-like morphology, and white arrows indicate M2-like morphology. (E) Immunofluorescence images showing MG-associated signals in the indicated groups (n=3). White arrows indicate extracellular MG. (F–J) Western blot analysis and quantification of M1-associated proteins, including CD86, iNOS, IL-1β, and TNF-α, in the indicated groups (n=3). (L) Representative fluorescence images showing intracellular ROS signals in the indicated groups (n=3). (M) Quantification of ROS fluorescence intensity using a fluorescence microplate reader (n=6). (N) Representative transmission electron microscopy images showing ultrastructural changes in HD11 cells in the indicated groups (n=3). Data are presented as means ± SD from at least three independent experiments. Different lowercase letters indicate statistically significant differences among groups (P < 0.05).


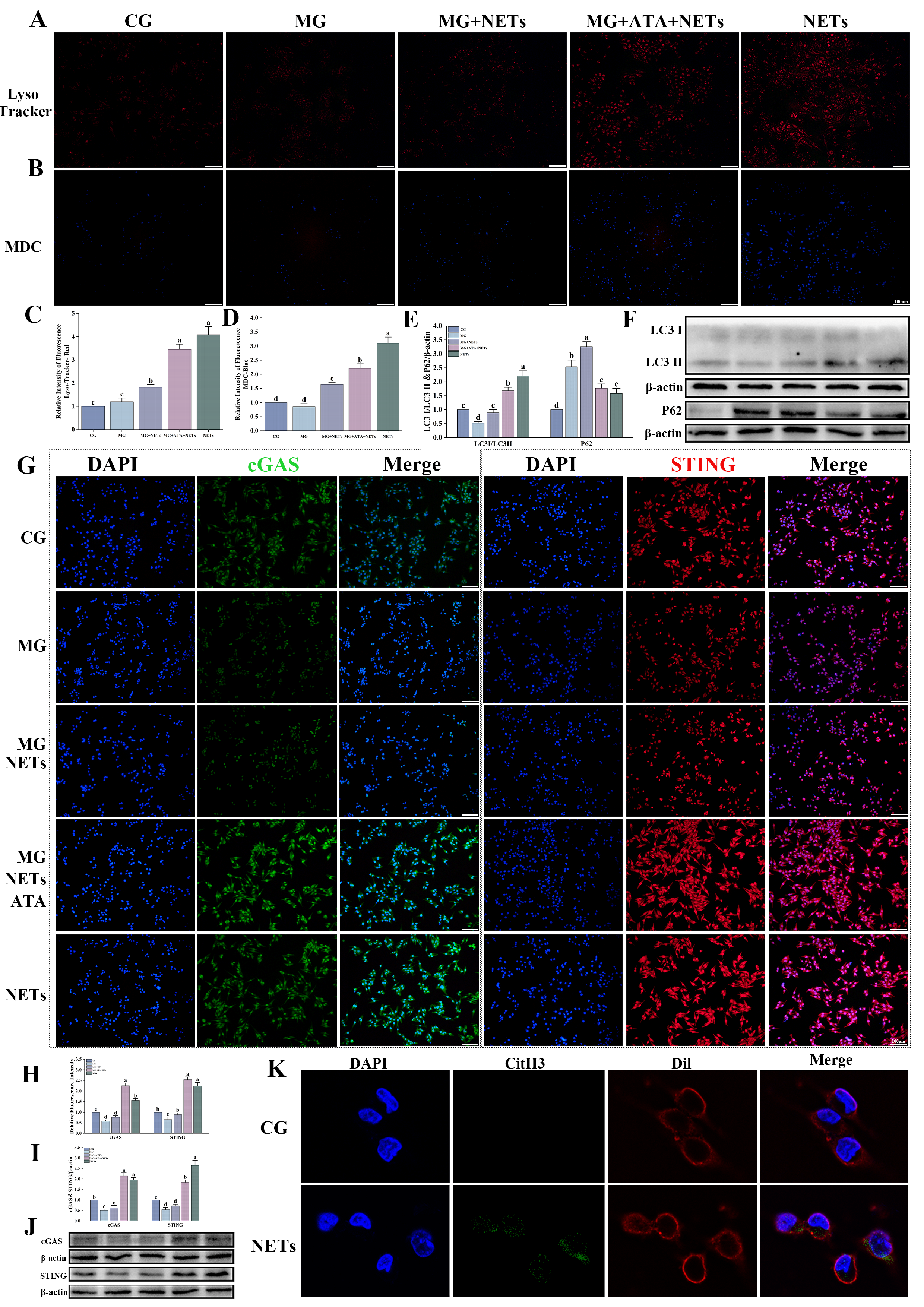


**Fig. S9. NETs protected from MG-mediated degradation are associated with autophagy-related changes and activation of cGAS/STING signaling in HD11 cells.** The grouping design used in this experiment was the same as that in Fig. S8. Compared with the MG and MG + NETs groups, the MG + ATA + NETs and NETs groups showed increased lysosomal abundance and autophagosome accumulation in HD11 cells (A-D). Western blot analysis showed that, relative to the CG group, the MG group exhibited a reduced LC3II/LC3I ratio and increased P62 expression, suggesting altered autophagy-related protein turnover after 10 h of MG infection.. In contrast, this pattern was reversed in the MG + ATA + NETs and NETs groups (E-F). In addition, confocal microscopy showed intracellular colocalization of NET-associated signals with HD11 cells after co-incubation (K). Because NETs are DNA-rich structures, the DNA-sensing cGAS/STING pathway was further examined. Compared with the MG and MG + NETs groups, NETs protected from MG-mediated degradation significantly increased cGAS- and STING-associated fluorescence signals and protein expression levels in HD11 cells (G-J).

(A, C) Lysosomes in HD11 cells were labeled with Lyso-Tracker Red (C1046, Beyotime) according to the manufacturer’s instructions, and representative fluorescence images and quantification of fluorescence intensity are shown (n=3). (B, D) Autophagosomes in HD11 cells were labeled with the MDC probe using an autophagy staining kit (C3018S, Beyotime), and representative fluorescence images and quantification are shown (n=3). (E, F) Western blot analysis and quantification of autophagy-related proteins LC3 and P62 in the indicated groups (n=3). (G, H) Representative fluorescence images and quantification of cGAS- and STING-associated signals in HD11 cells from the indicated groups (n=3). (I, J) Western blot analysis and quantification of cGAS- and STING-related protein expression in the indicated groups (n=3). (K) Representative confocal images showing colocalization of NET-associated signals with HD11 cells after co-incubation (n=3). NETs were labeled using the NET-associated marker CitH3. Data are presented as means ± SD from at least three independent experiments. Different lowercase letters indicate statistically significant differences among groups (P < 0.05).


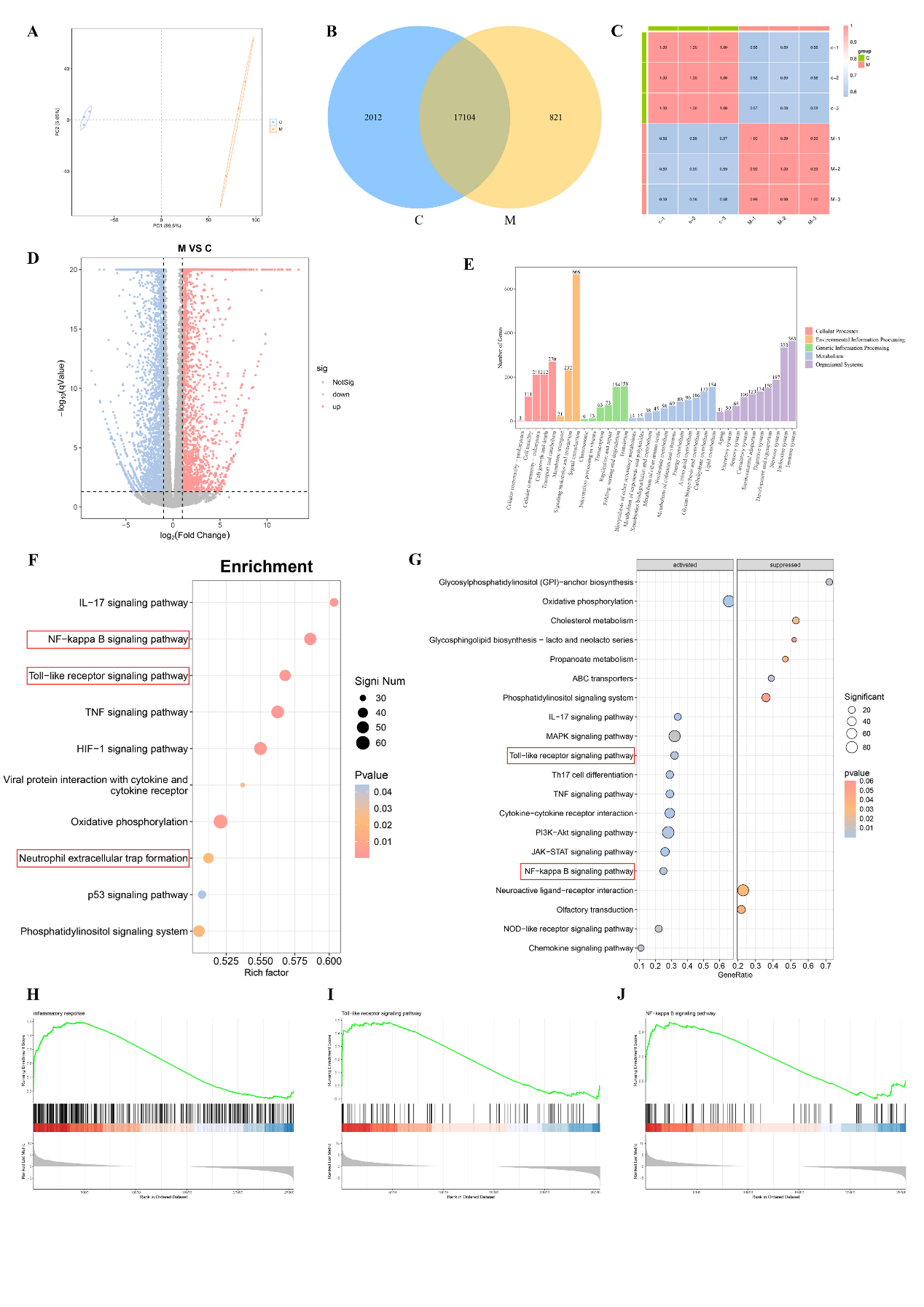


**Fig. S10. Transcriptomic analysis of immortalized chicken embryo tracheal epithelial cells (CETs) after MG infection.** To investigate the interaction between MG and tracheal epithelial cells, CETs were infected with MG (300 MOI) for 12 h and then subjected to transcriptomic analysis. Cells were divided into two groups (n=3): CG and MG. Compared with the CG group, MG infection induced differential expression of 7094 genes, including 3596 up-regulated and 3498 down-regulated genes (D). Gene Ontology (GO) classification analysis showed that the differentially expressed genes were mainly enriched in pathways related to signal transduction (Environmental Information Processing), immune system (Organismal Systems), endocrine system (Organismal Systems), transport and catabolism (Cellular Processes), and cell growth and death (Cellular Processes) (E). KEGG pathway analysis further showed enrichment of IL-17 signaling, NF-κB signaling, and Toll-like receptor signaling, and also suggested a close association between MG–CET interaction and pathways relevant to NET release (F). Gene set enrichment analysis (GSEA) further identified significant enrichment of classical MG infection-related pathways, including NF-κB signaling, Toll-like receptor signaling, MAPK signaling, PI3K/AKT signaling, and NOD-like receptor signaling (G). In addition, GSEA demonstrated significant enrichment of the inflammatory response pathway, Toll-like receptor signaling pathway, and NF-κB signaling pathway (H–J).

(A) Principal component analysis (PCA) showing transcriptomic differences between groups. (B) Venn diagram of expressed genes in the CG and MG groups. (C) Heatmap showing sample-to-sample correlation. (D) Volcano plot of differentially expressed genes between the CG and MG groups. (E) Bar plot showing GO functional classification of differentially expressed genes. (F) Bubble plot of KEGG pathway enrichment analysis. (G) Bubble plot of GSEA-enriched pathways. (H–J) GSEA plots showing enrichment of the inflammatory response pathway, Toll-like receptor signaling pathway, and NF-κB signaling pathway.


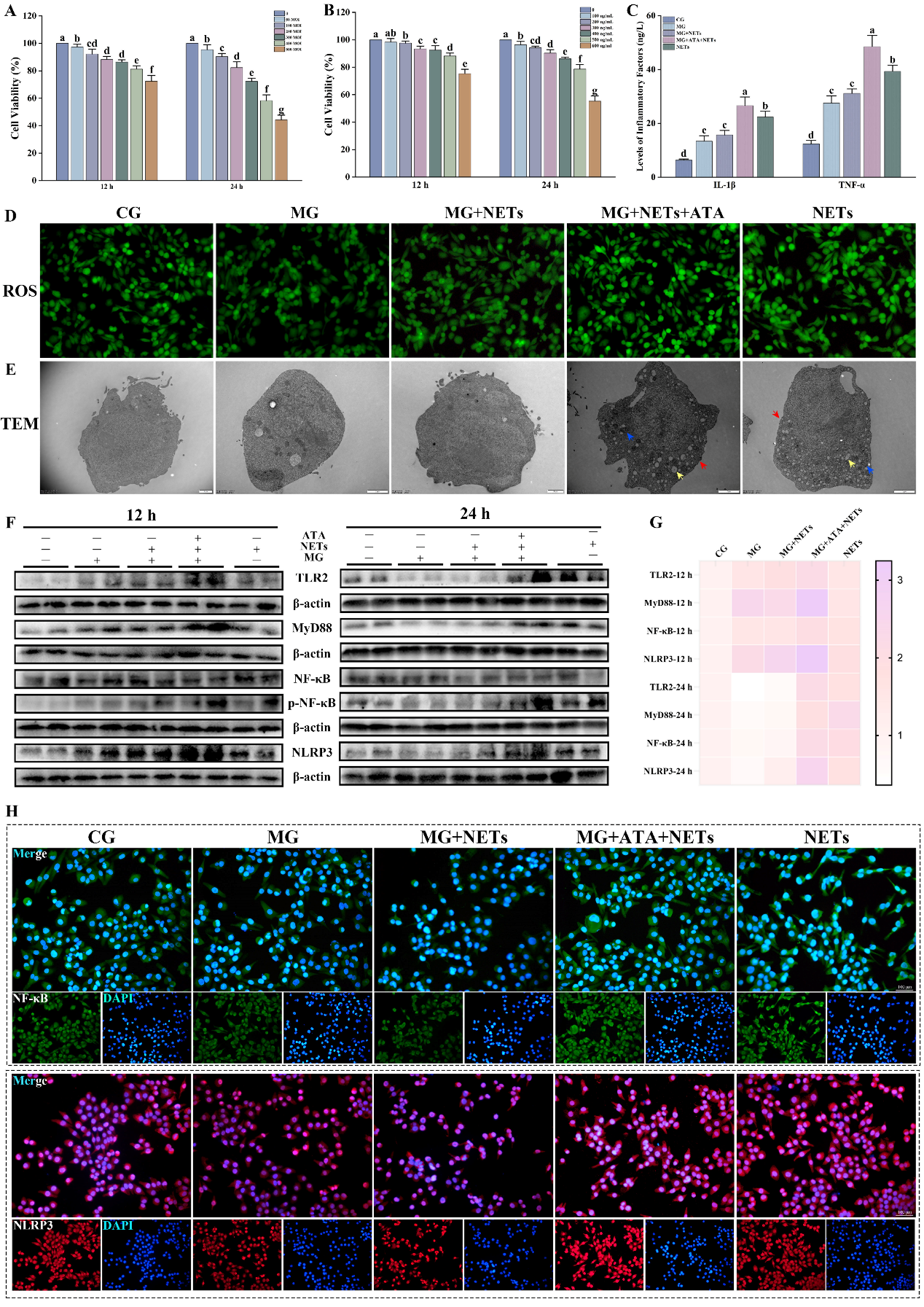


**Fig. S11. NETs protected from MG-mediated degradation sustain activation of TLR2/MyD88/NF-κB/NLRP3-associated signaling in CETs.** To evaluate the immunostimulatory effects of NETs on CETs, cells were treated with MG (300 MOI) and collected at 12 h or 24 h for subsequent analyses. A CCK-8 assay was first used to determine the working concentrations of MG and NETs, which were established as 300 MOI and 400 ng/mL, respectively (A, B). CETs were then divided into five groups: CG, MG, MG + NETs, MG + ATA + NETs, and NETs. Compared with the MG and MG + NETs groups, NETs protected from MG-mediated degradation significantly increased IL-1β and TNF-α levels in culture supernatants and enhanced intracellular ROS signals (C, D). Transmission electron microscopy showed that NET-treated CETs displayed swollen mitochondria, disrupted mitochondrial structure, increased cytoplasmic vacuolization, and roughened plasma membrane edges, suggesting ultrastructural changes associated with inflammatory cell death (E). Western blot analysis showed that, relative to the CG group, the MG and MG + NETs groups activated TLR2/MyD88/NF-κB/NLRP3-associated signaling at 12 h but showed a suppressed trend at 24 h. In contrast, the MG + ATA + NETs and NETs groups sustained increased expression of pathway-related proteins at both 12 h and 24 h (F, G). In addition, at 24 h, NETs protected from MG-mediated degradation markedly enhanced NF-κB and NLRP3 fluorescence signals in CETs (H). These findings indicate that intact NETs can sustain strong inflammatory activation in CETs.

(A-B) CCK-8 assay showing viability of CETs treated with different concentrations of MG or NETs for 12 h or 24 h (n=6). (C) ELISA analysis of IL-1β and TNF-α levels in culture supernatants from the indicated groups at 12 h and 24 h (n=6). (D) Representative fluorescence images showing intracellular ROS signals in CETs from the indicated groups (n=3). (E) Representative transmission electron microscopy images showing ultrastructural changes in CETs from the indicated groups (n=3). (F-G) Western blot analysis and quantification of TLR2/MyD88/NF-κB/NLRP3 pathway-related proteins in the indicated groups (n=4). (H) Representative immunofluorescence images showing NF-κB and NLRP3 signals in CETs from the indicated groups at 24 h (n=3). Data are presented as means ± SD from at least three independent experiments. Different lowercase letters indicate statistically significant differences among groups (P < 0.05).


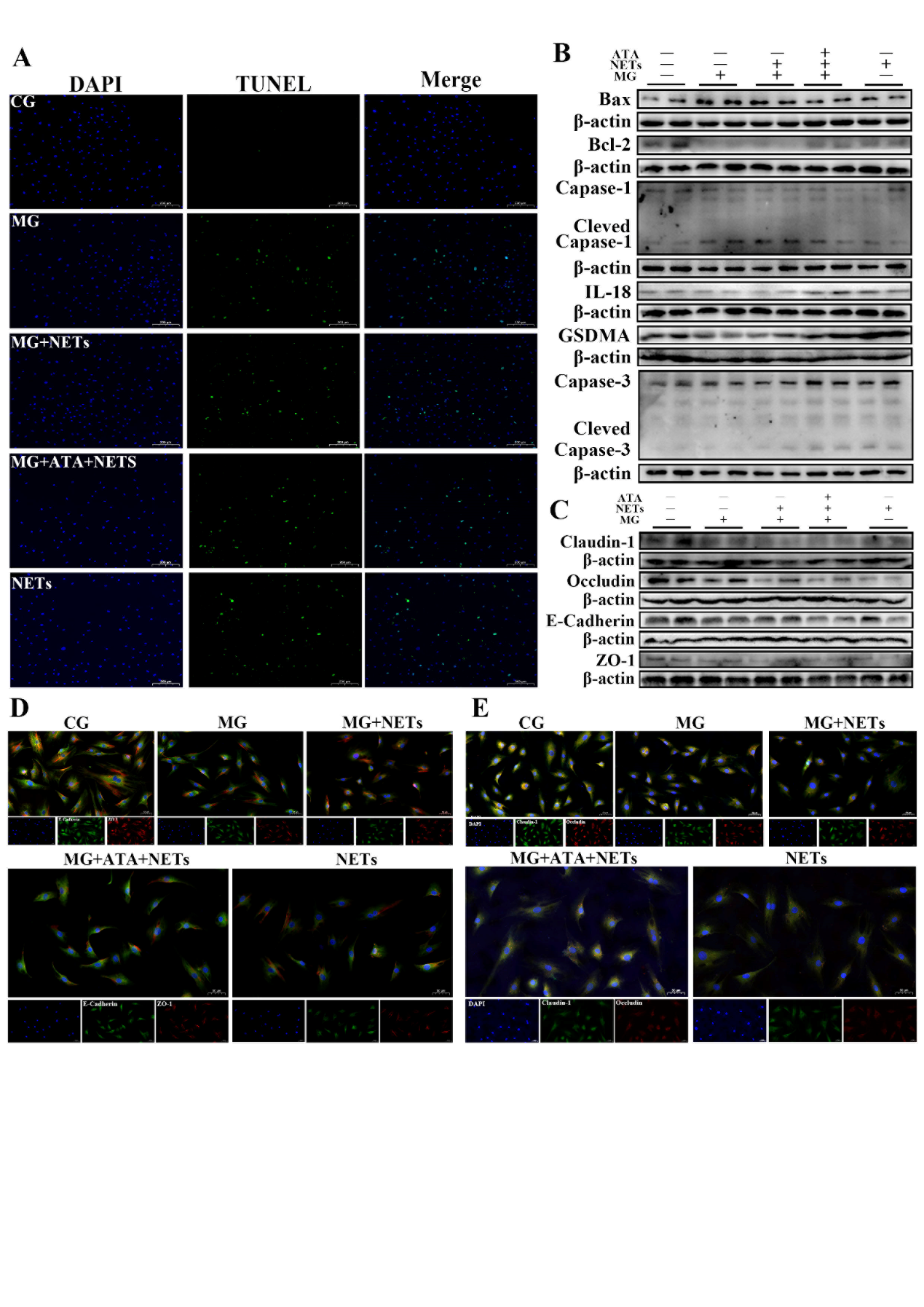


**Fig. S12. NETs protected from MG-mediated degradation are associated with inflammatory cell death–related signaling and barrier disruption in CETs.** The grouping design used in this experiment was the same as that in Fig. S11. To evaluate cell death–related changes in CETs after MG and NET treatment, apoptosis was first assessed by TUNEL staining. Both MG and NET treatment for 24 h increased the number of TUNEL-positive CETs (A). Western blot analysis was then used to examine apoptosis- and inflammatory cell death–related proteins. The MG and MG + NETs groups showed increased Bax and reduced Bcl-2 expression, consistent with apoptosis-associated changes, together with altered Caspase-1 expression. In contrast, the MG + ATA + NETs and NETs groups showed increased IL-18 and GSDMA expression, suggesting enhanced pyroptosis-associated inflammatory signaling. In contrast, the MG + ATA + NETs and NETs groups markedly increased the expression of IL-18, GSDMA, and Caspase-3 (B). In addition, NETs protected from MG-mediated degradation significantly reduced the expression and fluorescence intensity of the tight junction–associated proteins Claudin-1, Occludin, E-Cadherin, and ZO-1 (C–E), indicating impaired epithelial barrier integrity. Together, these findings suggest that intact NETs enhance inflammatory cell death–associated signaling in CETs and are accompanied by disruption of epithelial barrier function.

(A) TUNEL staining showing apoptotic CETs in the indicated groups (n=3). TUNEL-positive cells are shown in green, with representative images and quantification. (B) Western blot analysis of apoptosis- and pyroptosis-related proteins (Bax, Bcl-2, Caspase-1, IL-18, GSDMA, and Caspase-3) in the indicated groups (n=4). (C) Western blot analysis of tight junction–associated proteins (Claudin-1, Occludin, E-Cadherin, and ZO-1) in the indicated groups (n=4). (D–E) Representative immunofluorescence images and quantification of tight junction–associated proteins (Claudin-1, Occludin, E-Cadherin, and ZO-1) in the indicated groups (n=3). Data are presented as means ± SD from at least three independent experiments. Different lowercase letters indicate statistically significant differences among groups (P < 0.05).


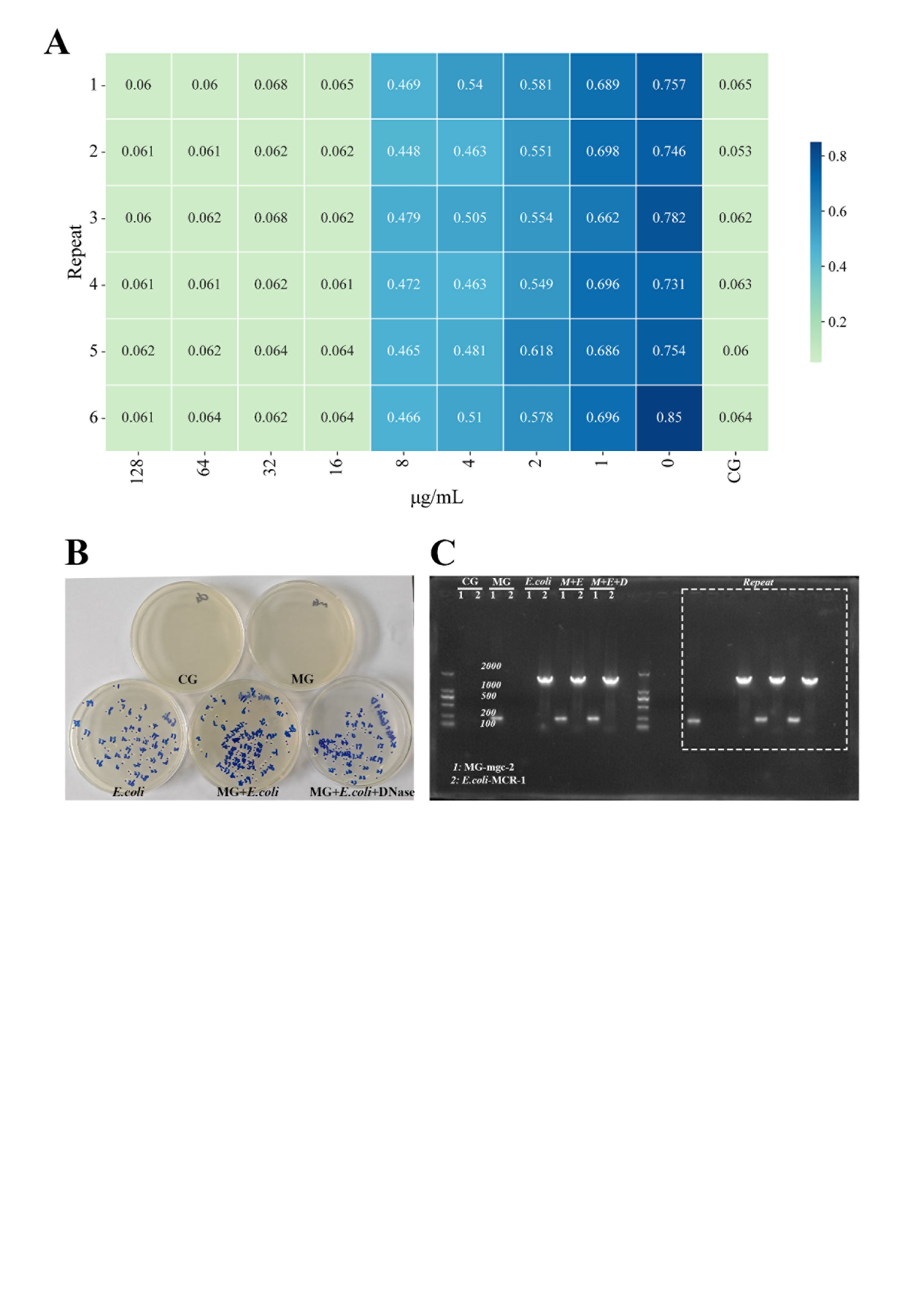


**Fig. S13. Establishment and validation of the chick co-infection model with MG and *E. coli* JD37.** *E. coli* JD37 was a pathogenic strain previously isolated in our laboratory from a farm in Harbin, China, where mixed infection with MG and *E. coli* had occurred, and was confirmed to be resistant to polymyxin E. (A) Minimum inhibitory concentration (MIC) of polymyxin E against *E. coli* JD37 (n=6). (B) Colon tissues from chicks in the indicated groups were homogenized in saline, serially diluted, plated on LB agar supplemented with polymyxin E (2 μg/mL), and incubated at 37°C for 12 h, after which colony numbers were recorded (n=3). (C) DNA extracted from tracheal and colon tissues of chicks in the indicated groups was used as template for PCR amplification of MG- or *E. coli* JD37-specific gene fragments, and the products were analyzed by 1% agarose gel electrophoresis (n=6).
